# ZBED6 Modulates the Transcription of Myogenic Genes in Mouse Myoblast Cells

**DOI:** 10.1371/journal.pone.0094187

**Published:** 2014-04-08

**Authors:** Lin Jiang, Ola Wallerman, Shady Younis, Carl-Johan Rubin, Elizabeth R. Gilbert, Elisabeth Sundström, Awaisa Ghazal, Xiaolan Zhang, Li Wang, Tarjei S. Mikkelsen, Göran Andersson, Leif Andersson

**Affiliations:** 1 Science for Life Laboratory, Department of Medical Biochemistry and Microbiology, Uppsala University, Uppsala, Sweden; 2 Department of Animal Production, Ain Shams University, Shoubra El-Kheima, Cairo, Egypt; 3 Department of Animal Breeding and Genetics, Swedish University of Agricultural Sciences, Uppsala, Sweden; 4 Broad Institute, Cambridge, Massachusetts, United States of America; 5 Harvard Stem Cell Institute and Department of Stem Cell and Regenerative Biology, Harvard University, Cambridge, Massachusetts, United States of America; Università degli Studi di Milano, Italy

## Abstract

ZBED6 is a recently discovered transcription factor, unique to placental mammals, that has evolved from a domesticated DNA transposon. It acts as a repressor at the *IGF2* locus. Here we show that ZBED6 acts as a transcriptional modulator in mouse myoblast cells, where more than 700 genes were differentially expressed after *Zbed6*-silencing. The most significantly enriched GO term was muscle protein and contractile fiber, which was consistent with increased myotube formation. Twenty small nucleolar RNAs all showed increased expression after *Zbed6*-silencing. The co-localization of histone marks and ZBED6 binding sites and the effect of *Zbed6*-silencing on distribution of histone marks was evaluated by ChIP-seq analysis. There was a strong association between ZBED6 binding sites and the H3K4me3, H3K4me2 and H3K27ac modifications, which are usually found at active promoters, but no association with the repressive mark H3K27me3. *Zbed6*-silencing led to increased enrichment of active marks at myogenic genes, in agreement with the RNA-seq findings. We propose that ZBED6 preferentially binds to active promoters and modulates transcriptional activity without recruiting repressive histone modifications.

## Introduction

ZBED6 was recently discovered as a novel transcriptional repressor of *IGF2* because a mutation disrupting one of its binding sites in porcine *IGF2* intron 3 leads to greater postnatal IGF2 expression in skeletal and cardiac muscle and makes pigs grow more muscle and a bigger heart [1], [2]. The ZBED6 protein contains two putative zinc-finger BED domains, named after the chromatin-boundary-element-binding proteins BEAF and DREF [3], and a hAT dimerization domain, a feature characteristic of the *hobo*-*Ac*-*Tam3* transposase superfamily [4]. ZBED6 is unique to placental mammals and has evolved from a domesticated DNA transposon. It appears to have evolved an essential function after the split between marsupials and placental mammals but before the radiation of placental mammals [1], [5]. The primary amino acid sequence of ZBED6, in particular the region comprising the DNA binding BED domains (residues 129–183 and 266–318), is highly conserved among all placental mammals for which sequence information is available (>26 species).

ZBED6 contains one nucleolar localization signal (residues 61–80), which targets ZBED6 protein into the nucleolus [1]. This lysine- and arginine-rich signal sequence is positively charged and extremely conserved among 26 placental mammals. This suggests that the nucleolar localization of ZBED6 is important for its function. The nucleolus is the site for ribosomal RNA (rRNA) synthesis, rRNA processing through small nucleolar ribonucleoproteins (snoRNPs) and ribosome assembly with ribosomal proteins [6]. A number of transcriptional regulators including MyoD and Myogenin repress rDNA transcription in the nucleolus during myogenesis of C2C12 cells [7].

ChIP-sequencing using mouse myoblast-derived C2C12 cells and an anti-ZBED6 antibody revealed approximately 2,500 putative ZBED6 binding sites [1], and 1,200 genes, including *Igf2*, contained one or several putative ZBED6 binding sites within 5 kb of the defined transcription start site (TSS). It remains to be shown to what extent ZBED6 regulates these genes and whether ZBED6 always acts as a repressor or if it functions as a transcriptional activator at some binding sites. Silencing of ZBED6 in C2C12 cells leads to an up-regulation of *Igf2* mRNA expression and enhanced cell growth, as well as enhanced myotube formation during differentiation [1]. Mild ZBED6 overexpression, on the other hand, leads to growth retardation in C2C12 cells [8].

The underlying pathways leading to changes in myotube formation, the mechanism for ZBED6-mediated transcriptional repression and the importance of ZBED6 in regulating targets other than *IGF2* are important questions that remain to be explored. Here, we used small interfering RNA (siRNA) to suppress *Zbed6* expression in mouse myoblasts, and studied the effect of silencing on global gene expression by RNA-seq and used microarrays to validate differential expression. Furthermore, we integrated expression data with ZBED6 ChIP-seq data, transcription factor binding, and performed ChIP-seq for histone modifications before and after silencing to investigate the regulatory mechanisms of *Zbed6*.

## Results

### *Zbed6-*silencing in mouse myoblast cells

**Figure 1:**
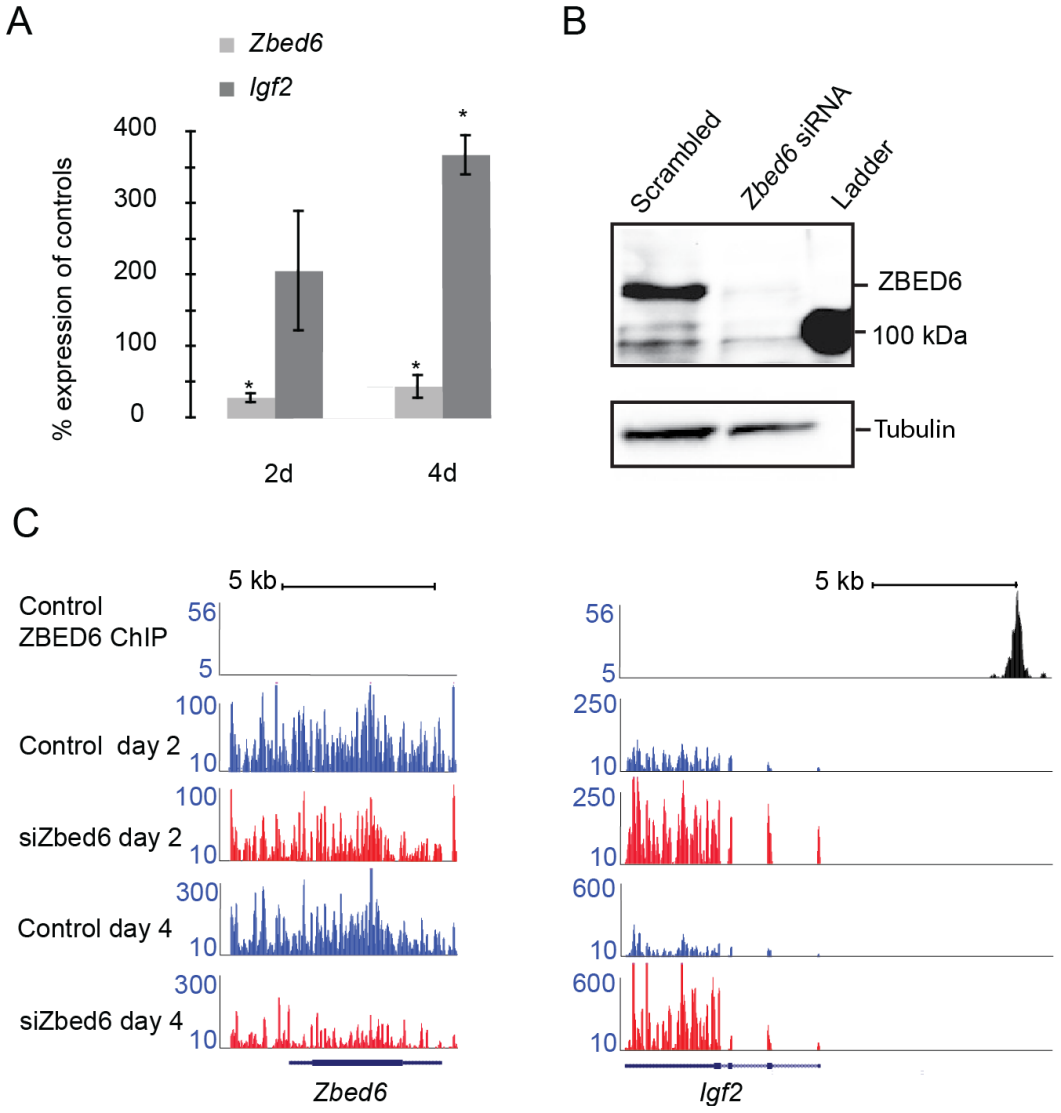
RNA sequencing of *Zbed6*-silenced myoblast cells. (A) Quantitative PCR validation of *Zbed6*-silencing (light gray bar) and *Igf2* up-regulation (dark gray bar) two and four days post-*Zbed6* siRNA transfection. Biological triplicates were performed for both *Zbed6* and scrambled siRNA. Error bars, s.e.m. The asterisk indicates a significant difference (P<0.05) between control and *Zbed6*-silenced samples. (B) Western blot validation of *Zbed6*-silencing. The protein lysates from the pooled biological triplicates for each siRNA treatment were equally loaded to the protein gel after measuring the concentration. The specific band for ZBED6 protein (110 kDa) appeared above the 100-kDa band from the protein ladder and α-Tubulin was used as a reference protein. (C) Direct comparison of the read counts (y-axis) from *Zbed6*-silenced and negative control RNA-seq data across *Zbed6* and *Igf2* regions for both two and four days post siRNA transfection. The ChIP-seq data with ZBED6 antibody from Markljung et al. [1] are shown to pinpoint the binding sites of ZBED6.

The *Zbed6*-silencing experiment was performed by transfecting C2C12 cells with a pool of *Zbed6* siRNAs. We used two different time points for analysis, two and four days post-transfection, and used a pool of scrambled siRNAs as a negative control. The silencing of *Zbed6* mRNA expression was verified by quantitative PCR. *Zbed6* mRNA was reduced by more than 70% and 50% on days 2 and 4, respectively (Figure 1A). ZBED6 protein level was efficiently suppressed on day 2, as verified by western blotting (Figure 1B). In addition, suppression of ZBED6 resulted in increased expression of its well-known target *Igf2*, starting on day 2 and reaching at least a 2.5- fold increase on day 4 (Figure 1A), consistent with our previous finding [1]. In summary, we efficiently silenced *Zbed6* in mouse myoblast cells and the level of silencing was sufficient to change the expression of downstream targets.

### Global effects of *Zbed6-*silencing measured by RNA-seq and expression arrays

We performed triplicate Zbed6 silencing experiments in C2C12 cells to prepare RNA for expression profiling with SOLiD strand-specific RNA sequencing and further used Illumina BeadChip arrays to validate the main results from the RNA-seq analysis. For RNA-seq, replicates were pooled and the sequencing gave 21–25 million uniquely aligned 50-bp reads per time point and sample (Table S1), of which 75% were mapped to known exons. By directly comparing read counts from the *Zbed6*-silenced samples with control samples across the *Zbed6* and *Igf2* genomic regions, we could verify a decreased expression of *Zbed6* and an increased expression of *Igf2* at both time points as expected (Figure 1C).

We calculated the gene expression in RPKM (reads per kilobase of exon per million mapped reads) using a gene model based on Ensembl annotations (see Methods). The detection threshold for the RNA-seq analysis was set to an RPKM value of at least 1 in at least one of the four samples. One RPKM has previously been estimated to equal one transcript per C2C12 cell [9]. We detected 13,344 (35.6%) out of the 37,515 annotated Ensembl genes in at least one sample using this criterion. Most genes were expressed with less than 100 copies per cell and only 82 (0.6%) expressed more than 500 copies per cell (Figure S1).

Differential expression (DE) between *Zbed6*-silenced and control samples was calculated using DEGseq [10] with a false discovery rate (FDR) of 0.001 and a fold change of 1.5 or higher. This gave 1,094 DE genes on day 2 and 4,412 on day 4. The correlation between RNA-seq fold changes computed on the data from day 2 versus that from day 4 was highly significant (r = 0.50, P<10^−16^, Figure S2A). Thus, most genes had the same direction of expression change from day 2 to day 4 after *Zbed6*-silencing. We reasoned that genes that show the same change of expression on both time points are less likely to be false positive and that the larger number of DE genes at the later timepoint indicates more secondary effects, thus we decided to focus on the DE genes at day 2 and also required the same direction of change between day 2 and day 4 for the definition of genes with significant differential expression. We thereby identified 780 DE genes after *Zbed6*-silencing in C2C12 cells.

In the microarray analysis, 8,537 (44.6%) out of 19,100 unique Ensembl genes present on the array were detected as being expressed. More than half of the DE genes (412/780) were either not identified as expressed or not present on the array. A large fraction (40%) of these genes belongs to different classes of non-coding RNAs, pseudogenes and hypothetical proteins that are not well represented on the array, and the remaining genes were on average more lowly expressed than the genes detected by both platforms (56% versus 69% had a RPKM<15, Table S2). The fold changes measured by RNA-seq and arrays were strongly correlated for the 368 DE genes that were detected with both methods (r = 0.56, P<0.001). About one third of these genes had a significant change in the same direction on the array (multiple test-adjusted P<0.05). The overlap of DE genes was lower for lowly expressed transcripts compared to those with medium or high expression (30% and 35%, respectively). This comparison strongly indicated that RNA-seq generated robust and reliable data and these have been used for the following analyses.

In total we found 20 small nucleolar RNA genes that were differentially expressed and strikingly, all of these genes were up-regulated after *Zbed6*-silencing at both time-points (Table 1). The up-regulation of the small nucleolar RNA genes was further confirmed by qPCR. Three of the five randomly selected genes showed up-regulation after *Zbed6*-silencing by qPCR and for two of these genes the change in RNA levels were statistically significant (P<0.05, Table 1).

**Table 1:** Small nucleolar RNA genes significantly up-regulated according to RNA-seq both on day 2 and day 4 after *Zbed6* silencing and qPCR validation.

Gene symbol	ZBED6 sites (within 10 kb)	Average expression	M-value		
			RNA-seq Day2	RNA-seq Day4	qPCR Day2
*Snord12*	no	high	1.22	1.05	−0.10
*Snord14c*	no	high	0.87	1.12	
*Snord14d*	no	high	0.85	0.80	
*Snord15a*	no	high	1.74	5.13	
*Snord21*	no	medium	1.29	1.70	
*Snord32a*	yes	high	0.64	1.40	
*Snord34*	yes	high	0.59	1.22	
*Snord35a*	yes	high	0.79	0.63	
*Snord35b*	no	high	1.48	1.02	
*Snord42a*	no	high	1.32	0.83	
*Snord47*	yes	high	1.01	0.89	0.14
*Snord49a*	no	high	0.73	1.46	
*Snord53*	no	high	1.33	0.56	
*Snord55*	no	high	0.98	1.26	
*Snord57*	yes	high	1.11	1.31	0.64^a^
*Snord65*	no	high	1.07	1.73	
*Snord66*	no	high	0.86	1.18	
*Snord82*	no	high	0.85	1.60	0.59^a^
*Snord95*	no	high	0.67	1.19	−0.28
*Snord100*	no	high	0.67	1.60	

a Student's t-test, P<0.05).

We further validated the expression changes for six protein-coding genes with low or medium expression (*Fgf11*, *Nfkbil1*, *Myo5a*, *Nr4a1*, *Sfrp2* and *Ddit4*) and one highly expressed long noncoding RNA (H19) by qPCR. The fold changes measured by RNA-seq and qPCR were significantly correlated (r = 0.84, P<0.001). All seven genes showed consistent direction of change between qPCR and RNA-seq, with significant qPCR values for all except *Ddit4* (Student's t-test, P<0.05, Table S3).

### Genes encoding muscle proteins were significantly over-represented among the DE genes

**Figure 2:**
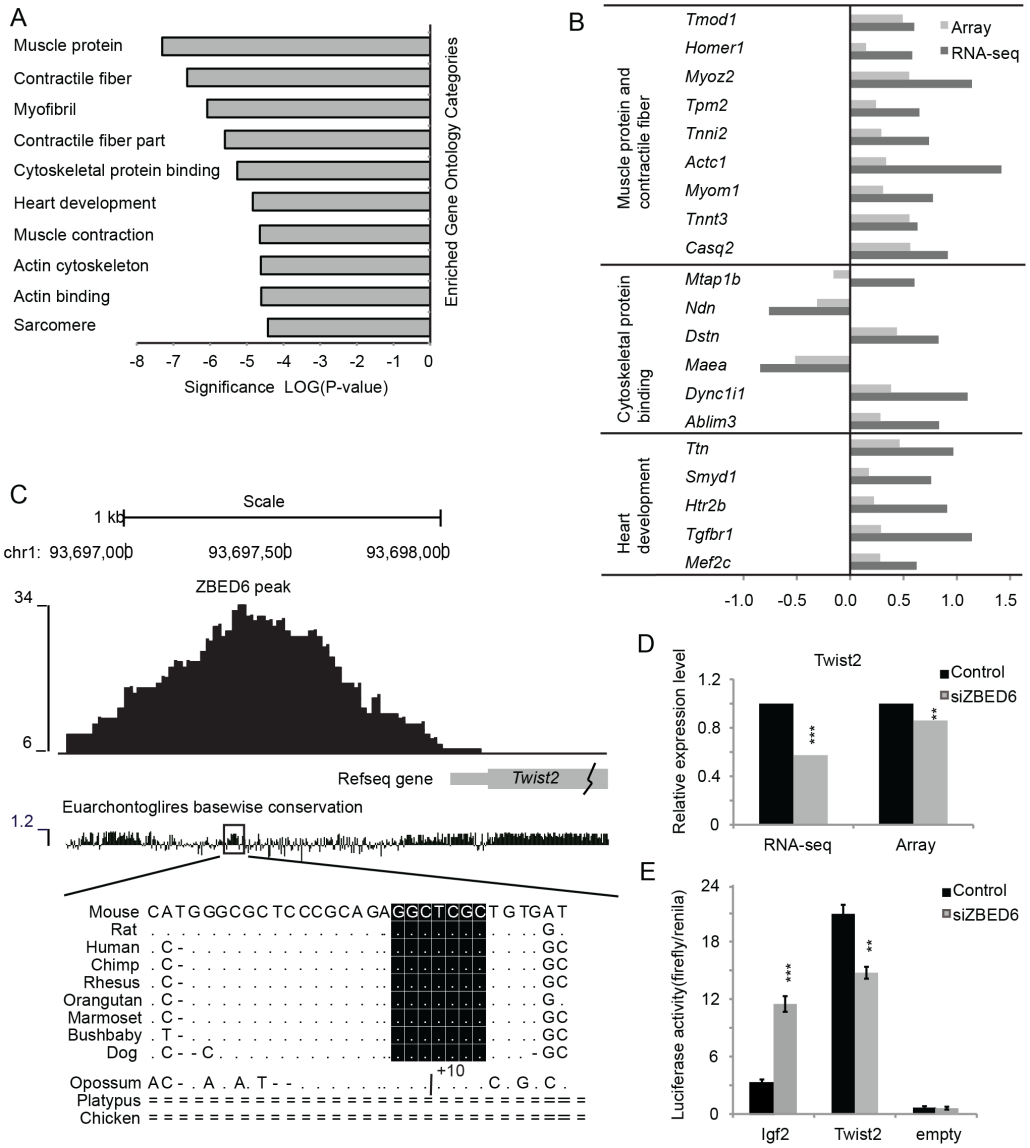
Muscle proteins were significantly enriched among the differentially expressed (DE) genes. (A) The significantly enriched GO categories (y-axis) in the DE genes with FDR <0.05. (B) The Log2 fold changes from both RNA-seq (dark) and arrays (grey) of the DE genes associated with muscle proteins and contractile fiber, cytoskeleton protein binding and heart development. (C) The ZBED6 peak at about 1 kb upstream of *Twist2* gene (grey box) displayed together with placental mammal conservation score. The peak maxima overlapped with a highly conserved region (grey box), which contained the consensus motif GCTCGC of ZBED6 only in the placental mammals. A 10-bp insertion (+10) was present within the consensus motif in the opossum genome, indicating lack of ZBED6 binding site in this region. (D) The relative expression levels of *Twist2* from RNA-seq and array in either *Zbed6*-silenced or control C2C12 cells. (E) The relative luciferase activity (the ratio of firefly to *Renilla*) of *Igf2*, *Twist2* and empty pGL3 basic constructs in *Zbed6*-silenced or control C2C12 cells. **, P<0.01; ***, P<0.001.

The 780 DE genes were submitted for an enrichment analysis of Gene Ontology terms and Kyoto Encyclopedia of Genes and Genomes (KEGG) pathways using the Database for Annotation, Visualization and Integrated Discovery (DAVID) (see Methods). We found highly significant enrichment for GO categories representing muscular functions, such as genes encoding muscle protein, contractile fiber, myofibril, heart development, muscle contraction, actin cytoskeleton, actin binding and sarcomere were significantly enriched (FDR-corrected P<0.05, Figure 2A, Table S4). A total of 64 genes were found in these GO categories, which could be further clustered into three groups (muscle protein and contractile fiber, cytoskeleton protein binding and heart development) and the RNA-seq data showed that the majority (54/64) were up-regulated after *Zbed6*-silencing. Forty of these genes were also present in the microarray analysis and half of them were identified as differentially expressed in both RNA-seq and array (Figure 2B). These include *Tmod1*, *Homer1*, *Myoz2*, *Tpm2*, *Tnni2*, *Actc1*, *Myom1*, *Tnnt3* and *Casq2*, all associated with muscle protein and contractile fiber, and all were significantly up-regulated after *Zbed6*-silencing. Moreover, genes associated with heart development (*Ttn*, *Smyd1*, *Htr2b*, *Tgfbr1* and *Mef2c*) were also up-regulated. For the genes associated with cytoskeleton protein binding, *Ndn* and *Maea* were down-regulated after silencing in both RNA-seq and array; whereas *Dstn*, *Dync1i1* and *Ablim3* were up-regulated after silencing. Only one gene (*Mtap1b*) showed a different direction of expression changes between RNA-seq and array data after silencing.

### Genes with ZBED6 binding sites are overrepresented among DE genes after *Zbed6*-silencing

In our previous C2C12 ChIP-seq experiment [1] we identified 2,500 putative ZBED6 binding sites, with close to 1,200 genes associated to ZBED6 peaks. Here, we performed a second ChIP-seq experiment using the Illumina HiSeq system to get a higher confidence in the localization of ZBED6 binding sites in C2C12 cells. We found a good agreement between the two replicates, but identified more peaks with better enrichment and resolution in the new dataset (Figure S3), and thus used the new dataset for comparison to differential expression. To estimate to what extent the loss of direct binding of ZBED6 influenced the expression of the target genes we first compared the new set of ZBED6 peaks (n = 3,780) to a filtered list of DE genes, where small non-coding RNAs and genes not present in the refGene dataset were removed. There was a significant enrichment for ZBED6 binding sites at DE genes compared to the expressed non-DE genes, and when the direction of change was considered this difference was found to come from up-regulated genes (Table 2) suggesting that ZBED6 primarily acts as a repressor.

**Table 2:** Analysis of the correlation between differential gene expression (DE) after *Zbed6* silencing and the presence of ZBED6 binding sites within 5 kb of the transcription start site (TSS).

		DE genes		
ZBED6 sites	Non DE genes	All	Up-regulated	Down-regulated
Yes	2681 (24.6%)	187 (29.8%)^a^	131 (32.8%)^a^	56 (24.6%)
No	8194	440	268	172
Total	10875	627	399	228

a Overrepresentation of genes with ZBED6 binding sites among DE genes (Chi square test, two-tailed, d.f. = 1; Non DE genes vs All DE genes, P = 0.004; Non DE genes vs up-regulated DE genes, P = 0.002)

The RNA-seq analysis at day 2 and 4 showed that there are three times more DE genes on day 4 than on day 2 but there was no significant overrepresentation of ZBED6 sites even for up-regulated DE genes on day 4 (27.2% versus 24.6% for all expressed genes, P = 0.25). This again suggests that there were more secondary effects on day 4.

Most of the DE genes were not identified as direct ZBED6 targets, which implies that the majority of differential expression is caused by secondary effects. We therefore searched the promoter region (from −2 kb to 2 kb of TSS) of the genes showing significant up- or down-regulation after *Zbed6*-silencing for overrepresentation of other transcription factor binding motifs using the oPOSSUM software (see Methods). There were five different motifs significantly overrepresented in up- and down-regulated genes (Table 3) and all five transcription factors (TFs) that bind to the corresponding motifs were expressed in C2C12 myoblast cells according to our RNA-seq data. Three of these transcription factor genes (*Nfkb1*, *Elk4*, and *Sp1*) were only overrepresented among down-regulated genes while the other two (*Mef2a* and *Prrx2*) were exclusive to up-regulated genes (Table 3). Of these genes, only SP1 had a significant ZBED6 ChIP-seq peak, but *Nfkb1* and *Elk4* had at least two-fold decrease in expression at day 2 after *Zbed6*-silencing according to qPCR analysis (Table 3).

**Table 3:** Overrepresentation analysis of transcription factor binding sites within 2- or downstream of the transcription start site of genes showing significant differential expression after *Zbed6*-silencing in C2C12 myoblast cells.

Transcription	TF	Up-/Down- regulation	Enrichment		Expression analysis	
factor	class		n^a^	P^b^	M^c^	P^d^
NFKB1	REL	down	125	1.54E-04	−2.03	0.01
ELK4	ETS	down	180	3.99E-04	−1.15	0.01
SP1	C2H2	down	318	3.24E-02	−0.21	0.54
MEF2A	MADS	up	98	1.27E-10	−0.58	0.22
PRXX2	HOMEO	up	186	4.72E-08	0.52	0.23

a Number of genes containing the transcription factor binding site.

b Fisher's exact test.

c M-values represent log2 transformation of fold changes between *Zbed6*-silenced samples and negative controls estimated by qPCR analysis. Triplicates for each treatment were used.

d Student's t-test were used to calculate the statistical significance.

### Validation of genes activated by ZBED6

The ZBED6 binding site (5′GGCTCGC3′) upstream of *Igf2* is 100% conserved among all placental mammals with sequence information. We therefore searched for other conserved matches to the core motif sequence in close vicinity to ZBED6 peaks located within 5 kb of genes. Overall, 22% of the peaks had a conserved GCTCG sequence located within 100 bp of the summit. For DE genes, we identified 45 genes that were associated with evolutionary conserved ZBED6 binding sites, with 17 down-regulated and 28 up-regulated after silencing ZBED6 (Table S5). There was a higher percentage of conserved sites among down-regulated genes (39% versus 27% for peaks with a motif match), however this difference did not reach significance (Fischer's exact test, P = 0.12). The up-regulation of *Igf2*, *Ppm1e*, *Igsf11*, *Ppm1l*, *Ablim3*, *G3bp2* and *Homer1* and the down-regulation of *Twist2*, *Sfrp2*, *Socs3* and *Slc9a3r1* were confirmed by array data. Interestingly, motifs in up-regulated ZBED6 target genes were associated with a palindrome structure that partially overlap the consensus motif, as is the case at the validated ZBED6 binding site in *Igf2*, while none of the down-regulated target genes were associated with a palindrome (Table S5).

To further explore the effect of the palindrome structure and if ZBED6 could also function as an activator, we analyzed a potential binding site 1 kb upstream of *Twist2,* a gene that encodes an important basic-helix-loop-helix transcriptional repressor of muscle-specific genes [11]. Similar to the *Igf2* binding site, this region has a ZBED6 consensus motif overlapping 16-bp that is highly conserved among all placental mammals (Figure 2C). The expression of *Twist2* was significantly down-regulated after *Zbed6*-silencing (Figure 2D), in contrast to the increased *Igf2* expression after *Zbed6*-silencing. We generated a construct with both the ZBED6 binding site and promoter from *Twist2* and a luciferase reporter gene cloned into a pGL3 basic vector. We transfected the construct into either *Zbed6*-silenced or control C2C12 cells and measured the luciferase activity; an empty pGL3 basic vector and a construct with the confirmed ZBED6 binding site from *Igf2* were used as negative and positive controls, respectively. The expression of the reporter gene after silencing was decreased using the *Twist2* construct but increased using the *Igf2* construct (Figure 2E), consistent with reduced *Twist2* expression and increased *Igf2* expression after *Zbed6*-silencing.

**Figure 3:**
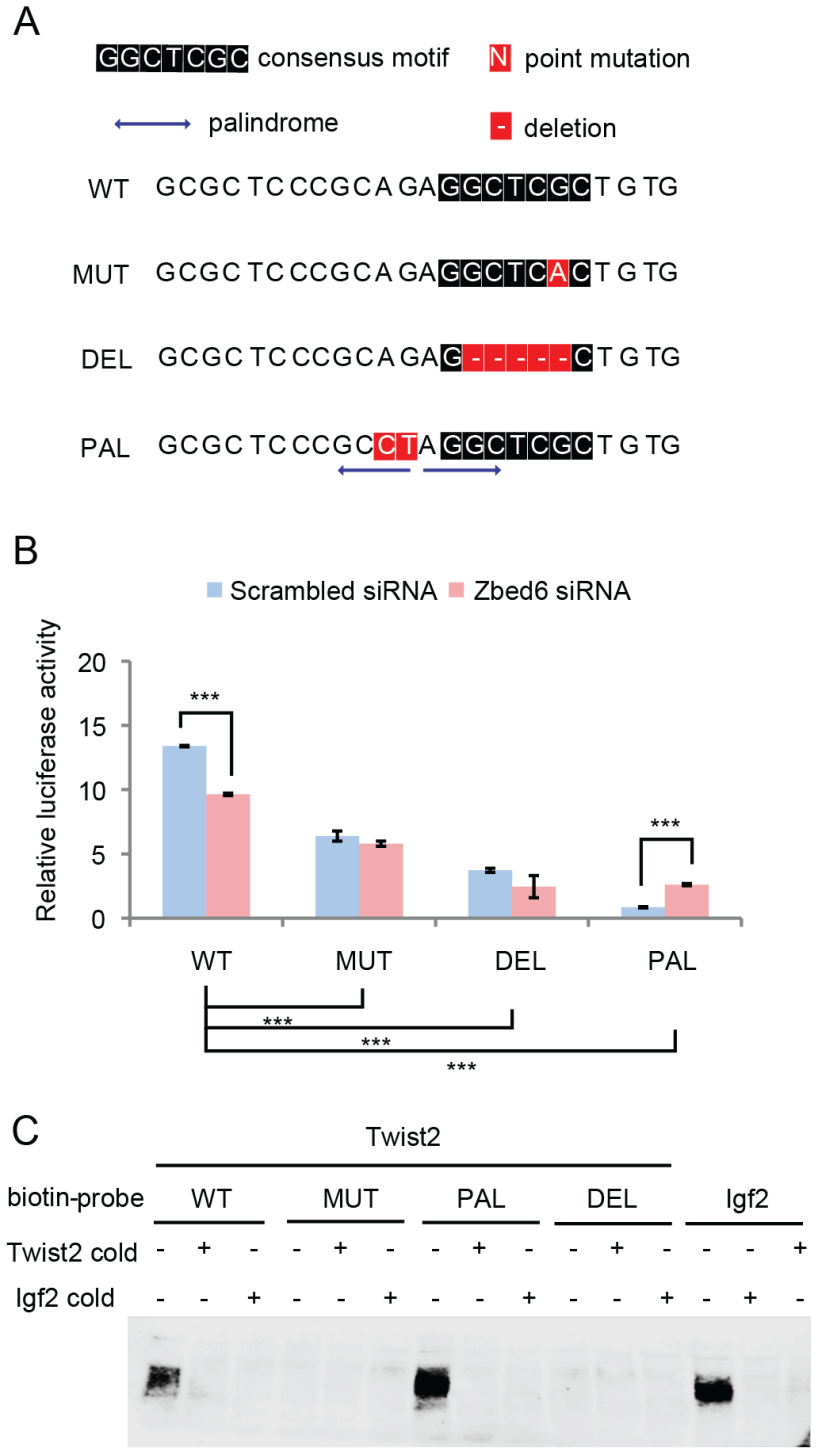
Luciferase assays for the *Twist2* locus using constructs where the ZBED6 binding site was altered. (A) The 24-bp conserved ZBED6 binding site at the *Twist2* gene (WT) was mutated to generate MUT containing a single G-to-A mutation at the ZBED6 consensus motif, to DEL by deleting the consensus motif, and to PAL by altering two nucleotides that created the same palindrome sequence as present at the *Igf2* locus (PAL). (B) The relative luciferase activity (the ratio of firefly to *Renilla*) of WT, MUT, DEL, PAL constructs in *Zbed6*-silenced (blue) or control C2C12 cells (pink). Standard errors of the mean are displayed by the error bars. ***, P<0.001. (C) EMSA of WT, MUT, DEL, PAL and *Igf2* biotin probes incubated with C2C12 nuclear extracts showing binding of endogenous ZBED6 to the WT, PAL and *Igf2* biotin probes but not to the MUT and DEL biotin-probes. The binding complex was competed using an 100-fold excess of the corresponding *Twist2* cold probe or *Igf2* cold probe.

To confirm that ZBED6 can activate *Twist2* transcription, we used the wild-type *Twist2* Luciferase construct (WT) and generated three different mutant constructs MUT, with a G-to-A mutation, DEL, with a deletion of the motif, and PAL that has the same palindrome sequence as present at the *Igf2* binding site (Figure 3A). After transfection of these four constructs into either *Zbed6*-silenced or control mouse myoblast cells, the luciferase activities were measured as before. Compared with the WT construct, both MUT and DEL reduced the expression of the reporter gene significantly (Figure 3B). Silencing of ZBED6 resulted in a significant decrease of the expression for the WT but not for the MUT and DEL constructs. These results imply that ZBED6 directly binds to the consensus motif upstream of *Twist2* and can activate transcription from the *Twist2* promoter, consistent with the RNA-seq data. Interestingly, the PAL construct that involves the same palindrome sequence as present at the *Igf2* binding site, showed a significant decrease in reporter gene expression compared with wild-type (Figure 3B). Furthermore, expression from the PAL construct increased after *Zbed6*-silencing (Figure 3B) implying that ZBED6 acts as a repressor in the context of the palindrome sequence. To further verify the direct binding of ZBED6 to the *Twist2* upstream region we carried out an electrophoretic mobility shift assay (EMSA) with wild-type (WT) and mutant oligonucleotides derived from MUT, DEL and PAL using nuclear extracts from mouse C2C12 myoblast cells (Figure 3C). The results indicated specific ZBED6-binding to the *Twist2* WT and PAL sequences but not to the MUT and DEL sequences in which the GCTCG consensus motif has been disrupted or deleted. Furthermore, the specificity was supported by the efficient competition achieved using a 50-fold excess of a cold Igf2-oligonucleotide containing a validated ZBED6 binding site. The new ChIP-seq dataset supports binding to *Twist2*, however, the signal over this region is relatively weak. To confirm the *in vivo*-binding to this region we performed ChIP-qPCR on a third sample, and further identified enrichment over the corresponding region in ChIP from human cell lines (Figure S4).

### ZBED6 binding sites are strongly associated with histone marks for active promoters

**Figure 4:**
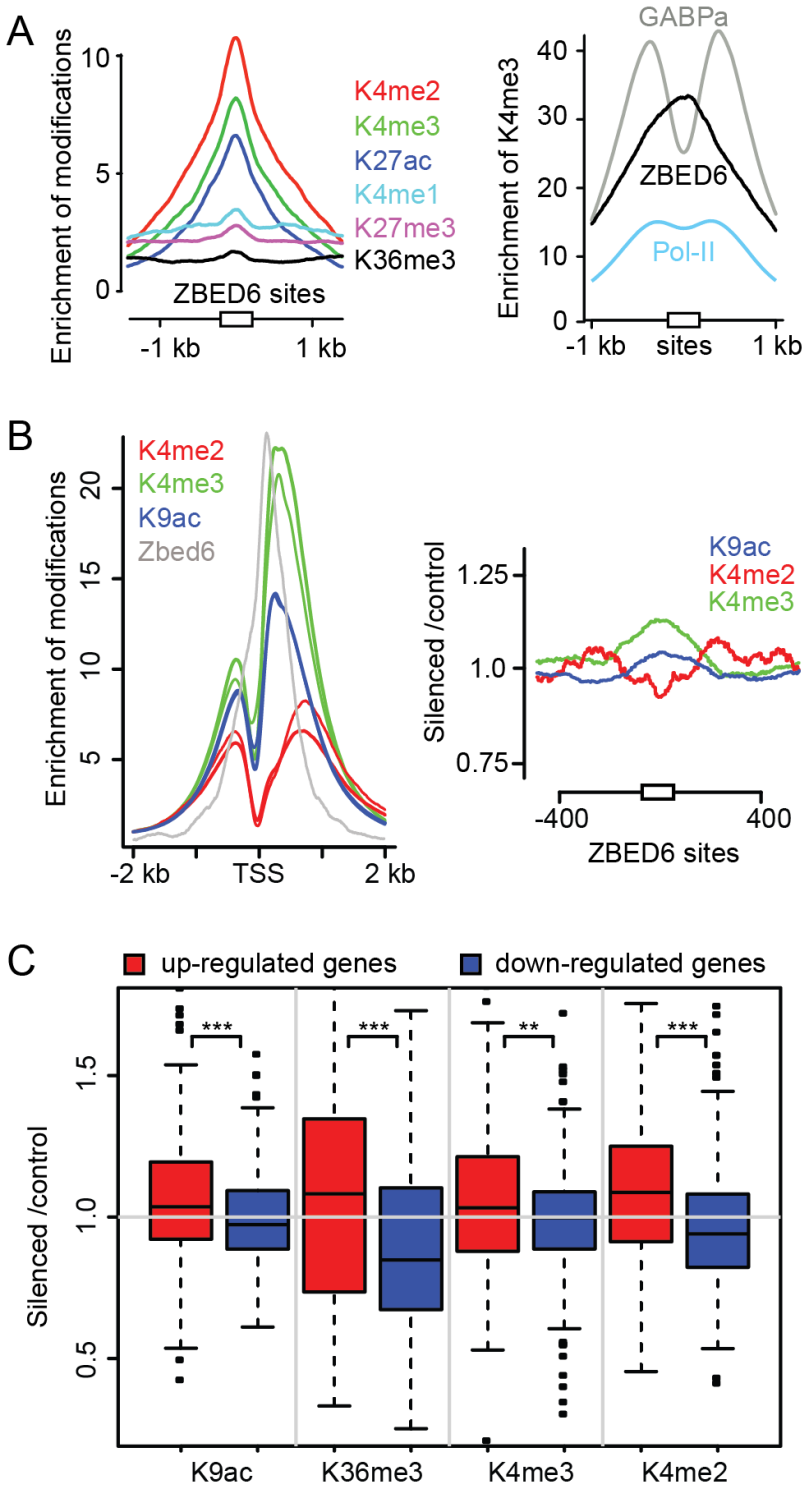
Histone modifications associated with ZBED6 target sites and other sites in C2C12 myoblast cells. (A) Left panel: ChIP sequencing data from six histone modifications including H3K4me2 (dark red), H3K4me3 (green), H3K27ac (purple), H3K4me1 (dark blue), H3K27me3 (pink), and H3K36me3 (black) located within 1 kb from ZBED6 target sites (white square). Right panel: enrichment of H3K4me3 over GABPa (grey), ZBED6 (dark) and Polymerase II (Pol-II) binding sites (light blue). (B) Left panel: genome-wide enrichment over TSS for active histone marks including H3k4me2 (dark red), H3K4me3 (green), H3K9ac (dark blue) in silenced (narrow lines) and control cells (bold lines). Signals for ZBED6 (dark) are shown for reference. Right panel: ratio of the enrichment between silenced and control (silenced/control) over ZBED6 peak centers for H3K4me2 (dark red), H3K4me3 (green) and H3K9ac (dark blue). (C) The histone modification ratios between silenced and control samples downstream of TSS for up-regulated and down-regulated (blue) genes after *Zbed6* silencing normalized to counts for all non-DE genes.

By overlapping the ChIP-seq signal of histone marks from untreated C2C12 myoblast cells (accession number GSE33227) with the ZBED6 binding sites, we observed a highly significant co-localization between ZBED6 and marks associated with open and active promoters (H3K4me2, H3K4me3 and H3K27ac; Figure 4A, left panel). No association was seen to marks found at enhancers (H3K4me1) or throughout expressed genes (H3K36me3), and further no enrichment was seen for genes which are in a silent state marked by repressive histone marks such as H3K27me3 (Figure 4A, left panel). This result was confirmed by analyzing a previously published dataset [12] on the distribution of histone marks in C2C12 cells. The average signal of histone modifications generally decrease over transcription factor binding sites [13] but for ZBED6 the signal peaked at the center of the ZBED6 binding sites, suggesting that it does not require histone-free DNA to bind. We compared the signal for H3K4me3 centered on the ZBED6 binding sequence GCTCG to that centered on peaks for a transcription factor (GABPa) known to bind in the nucleosome-free region upstream of TSS of active genes [14] and to that of PolII, which show a distribution similar to ZBED6 (Figure 3A, right panel). We further divided the ZBED6 binding sites into two groups, those associated with genes showing at least two-fold up-regulation after *Zbed6*-silencing and those at least two-fold down-regulated; only genes containing ZBED6 sites within 5 kb up- or downstream of TSS were included here. We observed similar profiles in the two groups for all six histone modifications except H3K4me2 (Figure S5). There was a stronger enrichment of the active histone mark H3K4me2 modification in the vicinity of ZBED6 binding sites of down-regulated genes after *Zbed6*-silencing (P<0.001, Student's t-test) compared with up- regulated genes.

### Histone modifications are differentially enriched at genes encoding muscle proteins after *Zbed6* silencing

To investigate whether silencing of ZBED6 causes a change in the histone marks, we performed ChIP sequencing on silenced and control cells with antibodies for the histone marks H3K4me3, H3K36me3, H3K4me2, and H3K9ac on day 4 after treatment (accession number SRA058767). The signal footprints over all TSS in the genome showed the expected pattern with a major peak downstream of TSS and an overall similar enrichment in silenced and control cells for the different modifications (Figure 4B, left panel). The combined ZBED6 signal has the maximum close to that for the active marks, 120 bp downstream of TSS. *Zbed6*-silencing had no major impact on the K4me2, K4me3 and K9ac signals over the ZBED6 binding sites as indicated by the silenced/control ratio for these histone marks (Figure 4B, right panel). We further analyzed the silenced/control ratios of K4me2, K4me3, K36me3 and K9ac reads in the first 2 kb downstream of TSS of all DE genes divided into up- and down-regulated genes. We observed a significantly higher enrichment for all modifications at up-regulated genes (Figure 4C), which further validates the RNA-seq results. Even though the DE genes had a significant change in the histone modification pattern as a group, many individual genes did not show any difference. We used the nine muscle related genes from the significant GO categories described above (*Tmod1*, *Homer1*, *Myoz2*, *Tpm2*, *Tnni2*, *Actc1*, *Myom1*, *Tnnt3* and *Casq2)*, which were identified as DE both by arrays and RNA-seq, as a set of true positive up-regulated genes and calculated the fold changes for these. The average fold changes for these genes were higher than the average for all up-regulated genes. H3k9ac showed the largest changes, with all nine genes above the average (median fold change = 1.33).

To test if the histone modification changes after *Zbed6* silencing supports the GO findings from the RNA-seq analysis categories, the 300 genes with the highest fold changes for H3K9ac was analyzed in the same way as DE genes using the DAVID software, with the genes expressed in C2C12 cells as the background. This gave highly significant enrichments for the same muscle-related categories as described above, with 17 of the 300 genes annotated as myofibril and contractile fibre (Table S6).

In conclusion, the data on histone marks suggest that ZBED6 interacts with active promoters and modulate their activity without recruiting major repressive histone modifications, and that the effect of *Zbed6* silencing is reflected in the histone modification pattern in agreement with the RNA-seq results.

### ZBED6 modulates the expression of *Igf2* and *Myogenin* during differentiation of C2C12 cells

**Figure 5:**
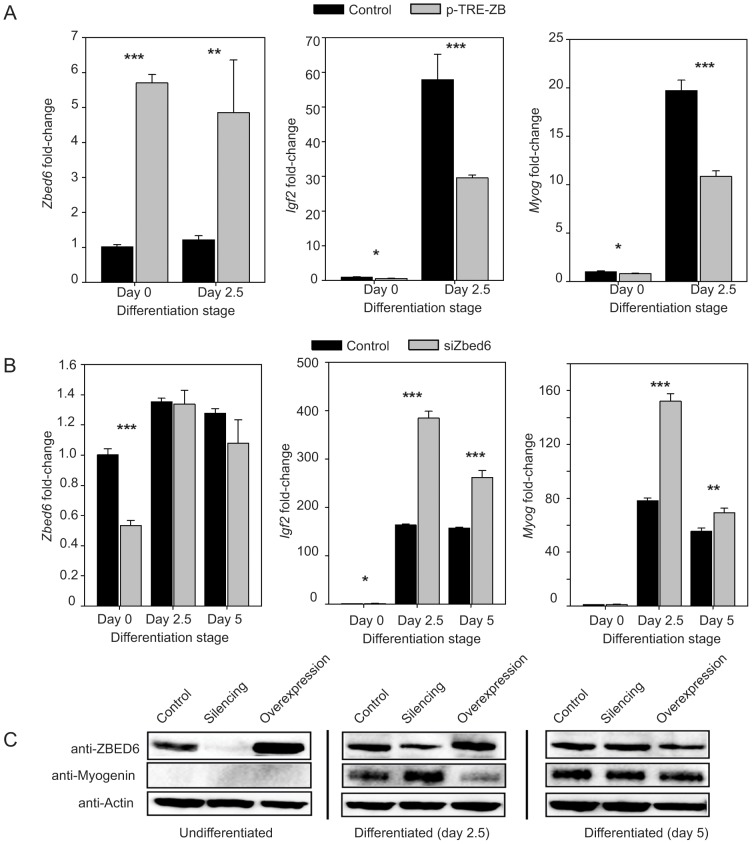
ZBED6 modulates the expression of Igf2 and Myogenin during differentiation of C2C12 cells. (A) Expression of *ZBED6*, *IGF2* and *Myogenin* mRNA monitored by qPCR before and after differentiation of control cells and transfected cells overexpressing ZBED6 from the pTRE-ZB vector. (B) Expression of *ZBED6*, *IGF2* and *Myogenin* mRNA monitored by qPCR before and after differentiation of control cells and cells in which ZBED6 has been silenced using siRNA. (C) Western blot analysis confirming altered expression of ZBED6 and Myogenin at the protein level. Actin was used as loading control.

C2C12 cells are differentiated towards myotubes when grown in low serum media. Myogenin (MYOG) is a muscle-specific transcription factor that is essential for myogenesis and shows together with IGF2 strongly induced expression during differentiation of C2C12 cells. We decided to explore how perturbed expression of ZBED6 affects *Igf2* and *Myogenin* expression during differentiation. Differentiation of C2C12 led to a minor (30–40%) increase in *ZBED6* expression (Figure 5). *Igf2* and *Myog* showed very low levels of expression in undifferentiated C2C12 but differentiation led to a dramatic increase in their expression (Figure 5A and 5B). Overexpression of ZBED6 resulted in a significantly reduced expression of *Igf2* and *Myog* both in differentiated and undifferentiated C2C12 cells (Figure 5A), with almost halved expression at day 2.5. Consistent with this result, *ZBED6* silencing resulted in increased expression of *Igf2* and *Myogenin* but this two-fold change was small compared with the dramatic change induced by differentiation (Figure 5B). The results demonstrate that ZBED6 does not determine if *Igf2* and *Myogenin* expression is on or off, but it modulates their level of expression after a developmental program has activated them.

## Discussion

The present study demonstrates that ZBED6 is an important transcriptional modulator since *Zbed6*-silencing in mouse C2C12 cells combined with transcriptome analysis revealed consistent changes in expression levels for more than 700 genes. Many of these transcriptional changes are likely to be secondary effects but the finding of a significant enrichment of differentially expressed genes associated with one or more ZBED6 binding sites, particularly for genes that are upregulated after ZBED6 silencing, strongly suggests that ZBED6 has many functional target sites in the genome besides the well-established and evolutionary conserved site in the third intron of *IGF2*. The finding of a fairly high proportion of differentially expressed genes lacking ZBED6 binding sites, implying that they represent secondary effects, was not unexpected since other transcription factor genes are highly enriched among the putative ZBED6 targets established by ChIP-seq analysis [1].

RNA-seq-based fold changes between *Zbed6*-silenced and control cells were significantly correlated with the fold changes measured by microarray hybridizations (r = 0.30, P<0.001) and the strength of correlation was similar to that observed in previous RNA-seq studies [15]. However, the correlation between the two platforms was weaker than the correlation between RNA-seq-derived fold changes from day 2 and day 4. One possible explanation for this is that we used a poly-A enrichment procedure for the RNA-seq experiment but not for the array experiment. Among the 780 DE genes identified by RNA-seq, we found a better correlation between RNA-seq and array data (r = 0.56, P<0.001) and at least one third of the genes that were identified as expressed in the array were validated as DE genes with the array. Especially for genes enriched in the top three Gene Ontology classifications (muscle protein and contractile fiber, heart development, and cytoskeleton protein binding, Figure 2B), all twenty genes except one had the same direction of fold changes between the two platforms, changes that all were statistically significant. The correlation between qPCR and RNA-seq fold changes of a small subset of genes was highly significant (r = 0.86, P<0.001) and eight out of twelve genes that were tested showed statistical significance using both methods. The excellent correlation observed for the 780 DE genes when comparing RNA-seq and microarray data across a large set of genes combined with qPCR data for a small set of genes confirm that our RNA-seq experimental design has generated reliable data for inferring differential gene expression between *Zbed6*-silenced cells versus untreated control cells.

The genes showing differential expression after *Zbed6*-silencing were significantly enriched for the GO terms muscle protein and contractile fibers (Table S4). The up-regulated genes encoding tropomodulin 1 (*Tmod1*), homer homolog 1 (*Homer1*), myozenin 2 (*Myoz2*), topomyosin 2 (*Tpm2*), skeletal troponins (*Tnni2* and *Tnnt3*), actin alpha cardiac muscle 1, myomesin 1 (*Mymo1*), and calsequestrin 1 (*Casq1*) constitute the majority of the contractile fiber category. These results are consistent with our previous observation that *Zbed6*-silencing resulted in increased myotube formation [1], because myotube formation requires the implementation of the contractile properties necessary for mature muscle fiber function [16]. However, only one of these nine genes, *Homer1* contains ZBED6 binding sites within 5 kb of TSS, indicating that the regulation of contractile fiber genes by ZBED6 is not direct but rather secondary effects on transcription probably regulated through other growth factors or transcription factors directly targeted by ZBED6. In agreement with this notion is that one growth factor (*Igf2*) and two myogenic transcription factors, myocyte enhancer factor 2c (*Mef2c*) and twist homolog 2 (*Twist2*), were differentially expressed after *Zbed6*-silencing. IGF2, an embryonic regulator of myogenesis and an autocrine factor that induces myoblast differentiation *in vitro* [17], is repressed by ZBED6 through a 5′GCTCG3′ consensus site. *Mef2c* is a well-known co-regulator for myogenesis [18] and it can activate the expression of downstream targets including muscle contraction genes [19]. *Mef2c* showed significantly greater expression after *Zbed6-*silencing, but there were no ZBED6 sites associated with this gene, thus the repression may not be through direct regulation. *Twist2*, which is known for its ability to inhibit myogenesis by repressing the transactivation activity of *Myod1* and *Mef2c* [11], was suppressed by the silencing of ZBED6 and contains an evolutionarily conserved ZBED6 binding site located immediately upstream of its TSS. Our Luciferase reporter assays and electrophoretic mobility shift assay suggested that *Twist2* may be a ZBED6 target. Therefore, ZBED6 may inhibit myogenesis through directly repressing *Igf2* and promoting *Twist2* expression.

A striking observation in the present study was the differential expression of genes encoding small nucleolar RNAs (snoRNAs). We found that the expression levels of 20 mouse snoRNAs identified by RNA-seq were significantly increased after *Zbed6*-silencing on both days. Two out of the five randomly selected snoRNAs were confirmed to be differentially expressed by qPCR validation. Interestingly, five out of the twenty snoRNAs contained ZBED6 binding sites within 10 kb of the TSS (Table 1). This indicates that ZBED6 directly or indirectly represses the transcription of these snoRNAs, which play important roles in pre-rRNA processing and modification in the nucleolus [20]. We previously showed that ZBED6 has both a nuclear and nucleolar localization sequence but has more intensive staining in the nucleolus versus nucleoplasma [1]. The functional significance of the nucleolar localization is not yet understood. Here we have discovered a potential role of *Zbed6* in regulation of snoRNAs expression in the nucleolus.

Does ZBED6 always act as a repressor? ZBED6 functions as a repressor of *Igf2 in vivo* by binding to a highly conserved sequence in the *IGF2* locus [1], [2]. Here, we found an enrichment for ZBED6 peaks at up-regulated genes which indicates that it is mainly a repressor. However, many down-regulated genes were associated to evolutionary conserved ZBED6 binding sites. A luciferase reporter assay supported the interpretation that *Twist2*, one of these down-regulated ZBED6 targets, is activated by ZBED6 through binding to its evolutionary conserved ZBED6 site. We found that a palindrome structure partially overlapping with the ZBED6 consensus motif was only associated with genes that were up-regulated after silencing suggesting that ZBED6 acts as a repressor at those sites. A previous study showed that mutations disrupting the palindrome structure at the *Igf2* QTN locus increased transcription of the reporter gene but did not affect ZBED6 binding [21]. Our EMSA and luciferase data (Figure 3) also showed that the palindrome structure that were created at the *Twist2* locus did not affect ZBED6 binding but switched the transcriptional activation to repression of the reporter gene. Thus, ZBED6 may act as a repressor if it binds to the consensus motif associated with a palindrome structure, while it may act as an activator in the absence of a palindrome. Furthermore, our previous *in vitro* DNA binding experiments showed that ZBED6 binding is sensitive to CpG methylation and only binds to non-methylated DNA [2]. The resulting chromatin structure obtained from CpG methylation and histone modifications, the presence or absence of the palindromic sequence and the functional interactions with other transcription factors that need to be present together with ZBED6 may determine whether ZBED6 can access a site and whether it acts as a transcriptional repressor or activator.

Our results reveal an emerging picture that ZBED6 tends to bind active promoters where it acts as a modulator of transcription rather than as a classical repressor or activator that acts via recruiting histone modifications. First, we found that ZBED6 sites are associated with high levels of H3K4me3, H3K4me2 and H3K27ac modifications, which are usually found at active promoters [22]–[24]. There was no enrichment of the repressive histone mark H3K27me3 at ZBED6 sites, which is consistent with our previous finding that ChIP-PCR did not reveal any change in the level of H3K27me3 modification at the *Igf2* locus after *Zbed6*-silencing [1]. Interestingly, a similar somewhat surprising association with active chromatin was recently detected for the SMRT corepressor [25]. Furthermore, our results when comparing undifferentiated and differentiated C2C12 cells (Figure 5) suggest that ZBED6 does not determine whether a gene is on or off but affects the level of expression after a developmental program has activated gene expression. This interpretation is fully consistent with the established role for ZBED6 in regulating *IGF2* expression in pig skeletal muscle. *IGF2* is expressed in wild-type pigs, which means that the promoter must be active, but *IGF2* is three-fold upregulated in mutant pigs, in which the ZBED6 binding site in *IGF2* intron 3 has been disrupted [2].

## Materials and Methods

### Cell culture

The C2C12 cells (ATCC, CRL-1772) used for these studies were between passages 10 and 18. Cells were maintained in Dulbecco's Modified Eagle's Medium (DMEM) with L-glutamine (ATCC, 30-2002), supplemented with 10% heat-inactivated fetal bovine serum and penicillin (0.2 U/mL)/streptomycin (0.2 μg/ml)/L-glutamine (0.2 μg/ml) (Gibco) at 37°C in a 5% CO2 humidified atmosphere. For the transfection experiments, cells were trypsinized at 70–80% confluence, and seeded at 100,000 cells per well in 12-well plates (Falcon).

### Differentiation of C2C12

Differentiation of C2C12 cells was induced by replacing the growth medium with a differentiation medium containing 2% horse serum. Total RNA and protein were extracted from the cells at the desired time point.

### Gene silencing using siRNA and validation

Cells were seeded in complete DMEM lacking antibiotics and reverse transfected with siRNA by lipofectamine 2000 (Invitrogen). *Zbed6*-silencing experiment and the validation of silencing with quantitative PCR and Western blotting followed the previous description [1]. Briefly, C2C12 cells were treated with *Zbed6* siRNA or scrambled siRNAs for two or four days in biological triplicates. Total RNA from each sample was extracted for qRT-PCR validation and microarray analysis. For qPCR validation, both 18S rRNA and beta-actin were used as endogenous controls. RNA-seq analysis used the pooled mRNA from the triplicates for each siRNA treatment. The protein lysates before and after two days *Zbed6*-silencing were prepared for western with anti-ZBED6 antibody and the α-Tubulin antibody (Cell signaling, #2144) as a reference.

The primer and probe sets for all qPCR reactions are listed in Table S7. All probes were TaqMan MGB probes with 5′-6FAM labels and 3′-non-fluorescent quenchers (Applied Biosystems). The qPCR validation of differentially expressed genes was done with the SYBR Green master mix (Applied Biosystems) according to the manufacturer's protocol. The fold-change expression was calculated by using the 2^−ΔΔCT^ method and Student's t-test was used to calculate the statistical significance.

The western and qPCR validation of the *Zbed6* silencing used for RNA-seq and array analysis were all based on the same siRNA experiment. The qPCR validation of differentially expressed genes identified by RNA-seq and all luciferase reporter assays were performed with new siRNA experiments.

### RNA sequencing analysis

Total RNA from C2C12 cells treated with scrambled siRNAs or *Zbed6* siRNAs two or four days post-transfection was isolated using the RNeasy mini kit (Qiagen). RNA triplicates from each treatment were pooled equally for mRNA enrichment by using the MicroPoly (A) Purist kit (Ambion). The quality and quantity of the mRNA was evaluated with a BioAnalyzer 2100 (Agilent). About 1 μg mRNA for each sample was used for RNA sequencing by AB SOLiD system (Life technologies). The fragment library was constructed according to the manufacturer's protocol (SOLiD Whole Transcriptome Analysis). Sequencing for each sample was done on a quarter of a slide and gave about 50 million 50 bp reads each. Wiggle files with read counts for genes were exported for UCSC browser to visualize the data. Ensembl gene model was modified to combine alternative transcripts into one gene. With the modified Ensembl gene model, we calculated gene expression by the reads per kilobase of gene model per million mapped reads (RPKM).

### Microarray analysis

All RNA samples used in array analyses had excellent quality according to Bioanalyzer. Biotinylated probes were prepared from 250 ng of total RNA, using the Ambion Illumina TotalPrep RNA Amplification kit (Applied Biosystems). The complete set of RNA was hybridized to MouseRef-8_v2 BeadChips (25K, Illumina). The raw data were processed using the software FlexArray (Version 1.4.1) [26], [27], which invokes R. The “lumi” algorithm bundle was chosen for data normalization.

### Significant differential gene expression

DEGseq [10] was used to identify differentially expressed genes from RNA-seq data, with random sampling model based on the reads count of each gene from *Zbed6*-silenced cells versus control cells. Statistical difference in gene expression for RNA-seq data was evaluated by the MA-plot-based method with random sampling model. M-values were determined by taking the log2 ratio of counts for silenced samples to that for controls. A-values were determined by taking the average log2 counts for silenced samples and controls. It was proven that under the random sampling assumption the conditional distribution of M-value given an observation of A-value follows an approximate normal distribution. The P-value was assigned based on the conditional normal distribution. Significant differential gene expression was determined using the following criteria: (i) less than 0.1% FDR, (ii) the same direction of fold change between day 2 and day 4, and (iii) at least 1.5 fold changes.

For the microarray data, statistical significance was evaluated using Bayes t-tests where three biological replicates of each treatment were contrasted against the opposite treatment at the same time point (the RNA-seq data was originated from an equal pooling of these triplicates). The P-values were corrected for multiple testing by using the Benjamini Hochberg approach [28]. The microarray criterion for DE was P<0.05 and the same direction of fold change between day 2 and day 4.

### Correlation analysis

For RNA-seq, microarray and qPCR analysis, the fold-change expression levels were calculated by the methods described in previous sections. The fold-change values were correlated and compared between platforms by using Pearson's correlation coefficient (r). The t-test was used to establish if the correlation coefficient is significantly different from zero.

### Gene Ontology analysis

The Ensembl Gene IDs of genes were submitted to the Database for Annotation, Visualization and Integrated Discovery (DAVID) Bioinformatics Resources 6.7 (http://david.abcc.ncifcrf.gov/) for the functional annotation chart analysis. The default setting was used for the GO analysis. We used the criteria of FDR-corrected P<0.05 and fold enrichment >2.5 to identify the significantly enriched GO categories.

### Overrepresentation of conserved transcription factor binding sites

To identify conserved ZBED6 binding sites, we use phastCons30wayPlacental scores of UCSC Genome Browser to measure conservation base by base. All ZBED6 peaks with a peak height of at least 12 were scanned for the presence of GTCTG sequences within 100 bp of the summit and the average conservation score was computed for each motif. The ZBED6 motif was considered conserved if the average conservation score was larger than 0.90.

To discover over-represented conserved transcription factor binding sites at the TSS of DE genes we used the web-based version 2.0 of oPOSSUM [29], [30]. Both up-regulated and down-regulated genes after *Zbed6-*silencing whether containing ZBED6 binding site or not were submitted for the module of Single Site Analysis. The software uses phylogenetic footprinting to reveal overrepresented matrices from the Jaspar database in foreground sequences. Statistical significance of results is measured by Fisher-score and Z-score and the significance criteria of Z-score >10 and Fisher score <0.05 were applied based on empirical studies. The Ensembl gene IDs for mouse were uploaded and scanned from −2 kb to +2 kb of the TSS against Jaspar vertebrate core profile. Level of conservation and matrix match threshold were kept at maximum. The over-represented transcription factors were sorted on bases of Fisher-score and overlapped with our RNA-seq data to filter out the factors that were not expressed in the C2C12 myoblast cells.

### DNA constructs for transfections

DNA constructs for the *IGF2* QTN were previously described [1]. Briefly, luciferase reporter plasmids contained 272 bp of the porcine *IGF2* P3 promoter, either alone or with either the wild type *IGF2* QTN region or mutant QTN region (581 bp insert) cloned directly upstream of the promoter. The difference between the wild type and mutant QTN is the substitution of an A for G at position 212 in the mutant construct. The *Twist2* pGL3 construct contained an 825 bp insert (chr1: 93697292-93698116, Mouse July 2007 (NCBI37/mm9) assembly) with two putative ZBED6 binding sites (starting pos. 93697347− strand and 93697464+ strand) and the *Twist2* promoter region cloned into the pGL3 basic vector (Promega). Constructs with the single mutation at the consensus motif (MUT), the deletion of the motif (DEL) and the creation of the palindrome sequence (PAL) in Figure 3 were generated with the QuickChange Site-Directed Mutagenesis kit according to the manufacturer's instructions (Agilent Technologies). In short, the mutations were made with ancestral *Twist2*-pGL3 construct as a template, and with oligonucleotides lacking the consensus motif or with point mutation(s). A synthetic vector was generated from the oligonucleotides annealed to the *Twist2*-pGL3 vector with *Pfu Turbo* polymerase (Agilent Technologies), and the ancestral vector was then digested with *DpnI*. The mutated constructs were sequenced to confirm the mutations. All DNA constructs were prepared by using EndoFree Plasmid Maxi kit (Qiagen).

### DNA transfections and reporter gene activity assays

After 24–48 h of incubation with siRNA treatments, C2C12 cells were washed and covered with Opti-MEM I Reduced Serum Media (Invitrogen). Cells were co-transfected with 1 μg of one endo-free DNA construct described in previous sections and 20 ng of *Renilla* vector, ph-RG-TK (Promega), using Opti-MEM I Reduced Serum Media and lipofectamine 2000 CD (Invitrogen). Cells were incubated for 24 h and luciferase activity was measured using the Dual Luciferase Reporter Assay Kit (Promega) and an Infinite M200 Luminometer (Tecan). Activity was expressed as relative luciferase units, the ratio of firefly luciferase activity to *Renilla* luciferase activity. Triplicates within an experiment were averaged. The experimental unit was defined as the experiment, with N = 4 experiments. Relative luciferase units were transformed (logX +1) to reduce heterogeneity of variance. Transformed data were analyzed in SigmaPlot v. 11.0 (Systat Software, Inc.) by two-way ANOVA. The statistical model included the main effects of siRNA treatment and DNA construct and the two-way interaction. Differences were considered significant at P<0.05. The Holm-Sidak method was used as a post-hoc test to evaluate all pairwise comparisons. Non-transformed data are shown graphically.

### Electrophoretic mobility shift assay (EMSA)

The ZBED6 binding sites in pig *IGF2* intron 3 and upstream mouse *Twist2* were used as probes to detect sequence-specific ZBED6 binding in vitro. The probes used were as follows: IGF2 wt, 5′-CTTCGCCTAGGCTCGCAGCGCGGGAGCGA-3′; Twist2 wt, 5′-TGGGCGCTCCCGCAGAGGCTCGCTGTGATGCCTAAGCT-3′; Twist2 mut, 5′-TGGGCGCTCCCGCAGAGGCTC**A**CTGTGATGCCTAAGCT-3′; Twist2 pal, 5′- TGGGCGCTCCCGC**CT**AGGCTCGCTGTGATGCCTAAGCT-3′; and Twist 2 del, 5′- TGGGCGCTCCCGCAGAGCTGTGATGCCTAAGCT-3′. ZBED6 binding sites are underlined, and altered sites are indicated in bold (Figure 3A). The probes were purchased 5′-Biotin labelled from Integrated DNA Technologies (Leuven, Belgium). Single-stranded complementary oligos were annealed in 1X NEB2 buffer (New England Biolabs) at 2 min at each degree from 95°C to 25°C to produce double-stranded probes. The binding reactions were then performed as previously described [1], with minor modifications. Briefly, a total of 3 μg C2C12 nuclear extracts was preincubated on ice for 20 min in binding buffer (kit specific binding buffer with supplements: 30.1 mM KCl, 2 mM MgCl2, 0.1 mM EDTA, 0.063% NP-40, 7.5% Glycerol, 1 μg/ml Poly(dI•dC)). Competition reactions were supplemented with 20 pmol (100-fold molar excess) unlabeled ds-oligonucleotide. After the addition of 200 fmol 5′-Biotin labeled ds-oligonucleotide, reactions were incubated at RT for 30 min. The protein–DNA complexes were separated on a 5% polyacrylamide gel (BioRad) run in 0.5×TBE at 100 V for 2:30 h in RT. Transfer to nylon membranes (Perkin Elmer) was carried out in 0.5×TBE at 45 V, 4°C for 1 h. The DNA was crosslinked for 15 min on a transilluminator with 312 nm bulbs, and then detected by the Lightshift Electrophoretic Mobility-Shift Assay kit (Thermo Scientific) by using streptavidin-horseradish peroxidase binding and chemiluminescent detection.

### ChIP-seq and histone modification analysis

Chromatin immunoprecipitation was done as previously described [1]. Briefly, C2C12 cells with and without *Zbed6* siRNA treatment were crosslinked using 1% formaldehyde for 10 min. Cells were lysed with cell lysis buffer and nuclei were resuspended in RIPA buffer and sonicated using a BioRuptor (Diagenode) to shear the chromatin. Antibodies for H3K4me3 (Millipore cs200554), H3K9Ac (Abcam ab4441) and H3k4me2 (Millipore 07-030) were bound to Protein-G magnetic beads (Invitrogen) and incubated with chromatin from 5-10 M cells per reaction at +4°C degrees for several hours. Beads were washed with RIPA and LiCl buffers and chromatin was eluted with SDS buffer. Indexed Illumina libraries were made using NEXTflex adaptors (BIOO Scientific) and sequenced to 50 bp on a HiSeq2000 instrument. Ampure XP (Beckman Coulter) was used to purify both eluted chromatin and the library reactions. The ZBED6 ChIP-seq experiment was done independently from the silencing experiment, with a higher cell count.

Reads were aligned to the mouse mm9 assembly using BWA with default parameters, and MACS [31] was used to identify peaks and create wiggle files with the signals. For histone modification enrichment analysis, BEDTools coverageBed [32] was used to count the number of uniquely aligned reads (mapping quality above 10) in a 2-kb window downstream of TSS. Only genes present in the refGene table (downloaded from the UCSC genome browser) was used. For genes with multiple TSS, only the TSS with the highest read count for H3K4me3 in the control sample was used for the analysis. The Mann-Whitney test was used to calculate significance of count differences.

ChIP-seq datasets for GABPa and PolII were downloaded from the ENCODE repository through the UCSC genome browser.

### Histone modification analysis over ZBED6 target sites

ChIP-sequencing reads for six histone modifications in C2C12 myoblast cells including H3K4me1, H3K4me2, H3K4me3, H3K27me3, H3K27ac and H3K36me3 were aligned to the Mouse genome July 2007 (NCBI37/mm9) assembly by using Mosaik (version 1.0.1384) with default parameters (http://bioinformatics.bc.edu/marthlab/Mosaik). The uniquely aligned reads were submitted for calling peaks by using Model-based analysis of ChIP-sequencing (MACS). We extracted the signals for each histone mark in a 3.5 kb window surrounding the center of the ZBED6 peak maxima and made the plot with the average overlapped reads for all six histone modifications. We did similar plots for both up- and down-regulated ZBED6 target genes after the silencing.

### Overexpression of ZBED6

The coding sequence of mouse ZBED6 was cloned directly downstream of Tetracycline-responsive promoter in pTRE-Tight vector (Clontech) between BamHI and SalI sites. The EndoFree Plasmid Maxi kit (Qiagen) was used to produce endotoxin-free pTRE-ZB construct. In the same time, Tet-On-Advanced vector (Clontech) was used to generate stable C2C12 cell line expressing Tet-controlled transactivator protein. Those stable cells were transit transfected with pTRE-ZB construct (1 μg/ml) using lipofectamin2000 (Invitrogen). Doxycycline (500 ng/ml) was added to the culture medium to induce the expression of ZBED6 from pTRE-ZB vector.

### Immunoblot analysis

Cells were washed with PBS and lysed in RIPA lysis buffer containing protease inhibitors (Roche). Lysates were vortexed and incubated on ice for 15 min and centrifuged at 13000 RPM for 15 min at 4°C. The supernatants were transferred into new tubes and the concentrations of the proteins were determined by Bradford protein assay (BioRad). Equal amounts of total lysates were separated by SDS-PAGE gels (4%–12%, Bio-Rad) and transferred into PVDF membranes (Millipore). Membrane was blocked by SuperBlock blocking buffer (Thermo scientific) and then incubated overnight at 4°C with diluted primary ZBED6 (1∶1000), Myogenin (1∶1000, SantaCruz) or Actin (1∶5000, Abcam) antibodies. Thereafter, the membrane was incubated with secondary anti-rabbit IgG or anti-mouse IgG (1∶5000, cell signaling) antibodies conjugated to horseradish-peroxidase (HRP). Proteins were visualized and detected by chemiluminescence ECL prime detection reagent (Amersham).

### Data access

The RNA-seq data from this study have been submitted to the NCBI Sequence Read Archive (http://trace.ncbi.nlm.nih.gov/Traces/sra/) under accession number SRA047361. The microarray data and the ChIP-sequencing data have been submitted to NCBI Gene Expression Omnibus (http://www.ncbi.nlm.nih.gov/geo/) under accession number GSE33430 and GSE33227.

## Supporting Information

Figure S1 **The distribution of the mRNA expression of the Ensembl genes measured by RPKM (reads per kilobase of gene model per million mapped reads).**
(PDF)

Figure S2 **Analysis of RNA-seq data.** A. The correlation between the Log2 RNA-seq fold changes computed on the data from day 4 (y-axis) versus that from day 2 (x-axis); B. The correlation between the Log2 fold changes measured by RNA-seq and microarrays for all genes.
(PDF)

Figure S3 **Results of ZBED6 ChIP-seq analysis in C2C12 cells.**
(PDF)

Figure S4 **Validation of ZBED6 binding to the conserved element upstream of** ***Twist2*** **.**
(PDF)

Figure S5 **Enrichment analysis of six histone modifications across ZBED6 target sites.** ZBED6 target genes were divided into up-regulated and down-regulated genes after Zbed6 silencing.
(PDF)

Table S1 **Summary of RNA sequencing and reads mapping.**
(PDF)

Table S2 **Differentially expressed genes identified by RNA-seq.**
(PDF)

Table S3 **qPCR validation of 12 differentially expressed genes that were identified by RNA-seq.**
(PDF)

Table S4 **Gene Ontology analysis of genes with differential expressions identified by RNA-seq.**
(PDF)

Table S5 **Twenty-six differentially expressed genes with evolutionary conserved ZBED6 binding sites.**
(PDF)

Table S6 **Significant GO categories for the top 300 H3K9ac genes.**
(PDF)

Table S7 **Primer and probe sets for qPCR validation.**
(PDF)
