# Supplementary material for: ZBED6 Modulates the Transcription of Myogenic Genes in Mouse Myoblast Cells
Source: PLoS One. 2014 Apr 8;9(4):e94187. doi: 10.1371/journal.pone.0094187 (PMC3979763; doi:10.1371/journal.pone.0094187)
Supplement: Figure S1 — The distribution of the mRNA expression of the Ensembl genes measured by RPKM (reads per kilobase of gene model per million mapped reads). (PDF) [file pone.0094187.s001.pdf]

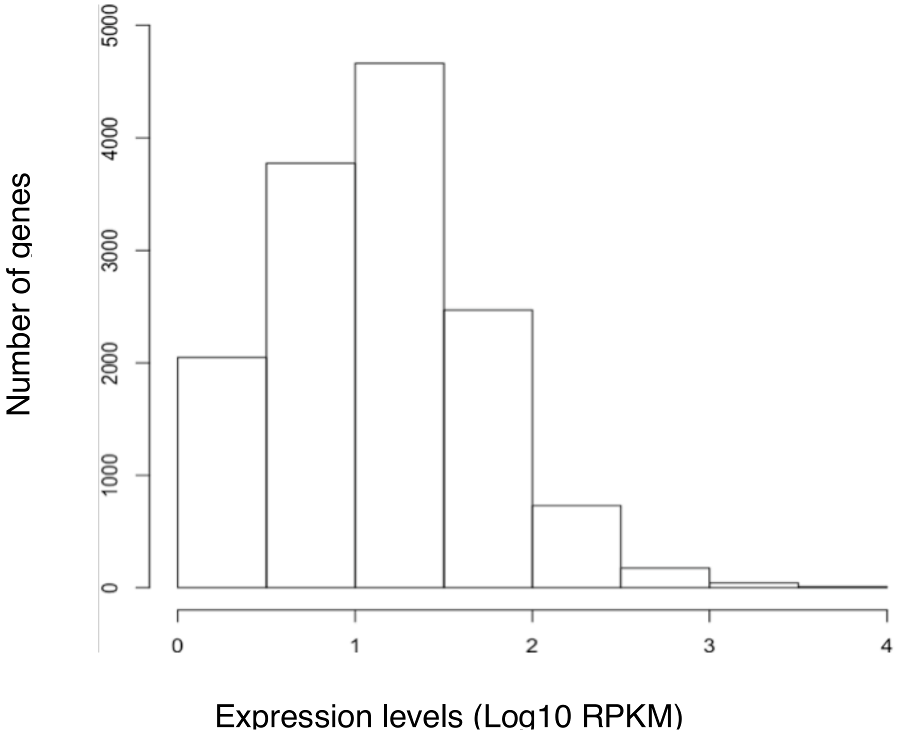

Figure S1. The distribution of the mRNA expression of the Ensembl genes measured by RPKM (reads per kilobase of gene model per million mapped reads).
