# Supplementary material for: ZBED6 Modulates the Transcription of Myogenic Genes in Mouse Myoblast Cells
Source: PLoS One. 2014 Apr 8;9(4):e94187. doi: 10.1371/journal.pone.0094187 (PMC3979763; doi:10.1371/journal.pone.0094187)
Supplement: Figure S2 — Analysis of RNA-seq data. A. The correlation between the Log2 RNA-seq fold changes computed on the data from day 4 (y-axis) versus that from day 2 (x-axis); B. The correlation between the Log2 fold changes measured by RNA-seq and microarrays for all genes. (PDF) [file pone.0094187.s002.pdf]

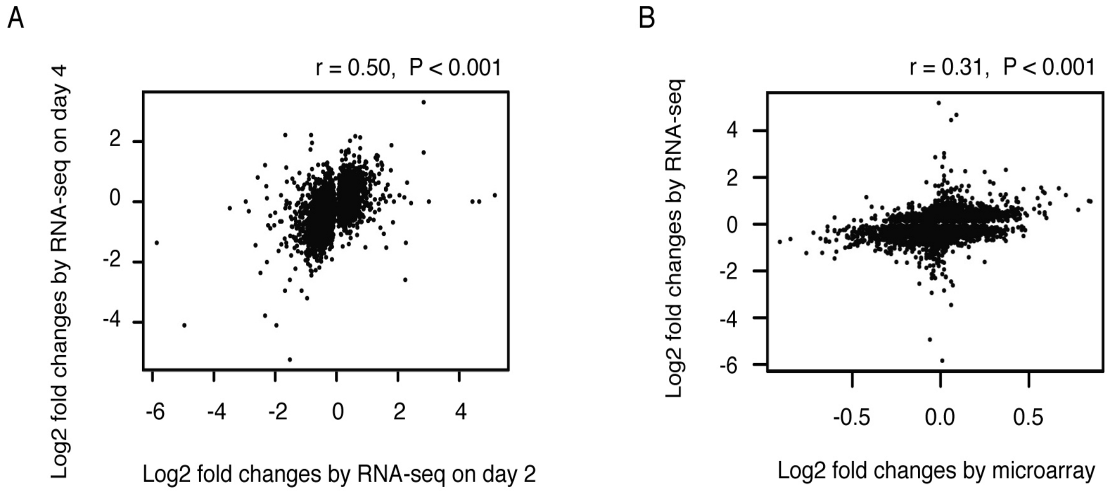

Figure S2. Analysis of RNA-seq data. A. The correlation between the Log2 RNA-seq fold changes computed on the data from day 4 (y-axis) versus that from day 2 (x-axis); B. The correlation between the Log2 fold changes measured by RNA-seq and microarrays for all genes.
