# Supplementary material for: ZBED6 Modulates the Transcription of Myogenic Genes in Mouse Myoblast Cells
Source: PLoS One. 2014 Apr 8;9(4):e94187. doi: 10.1371/journal.pone.0094187 (PMC3979763; doi:10.1371/journal.pone.0094187)
Supplement: Figure S3 — Results of ZBED6 ChIP-seq analysis in C2C12 cells. (PDF) [file pone.0094187.s003.pdf]

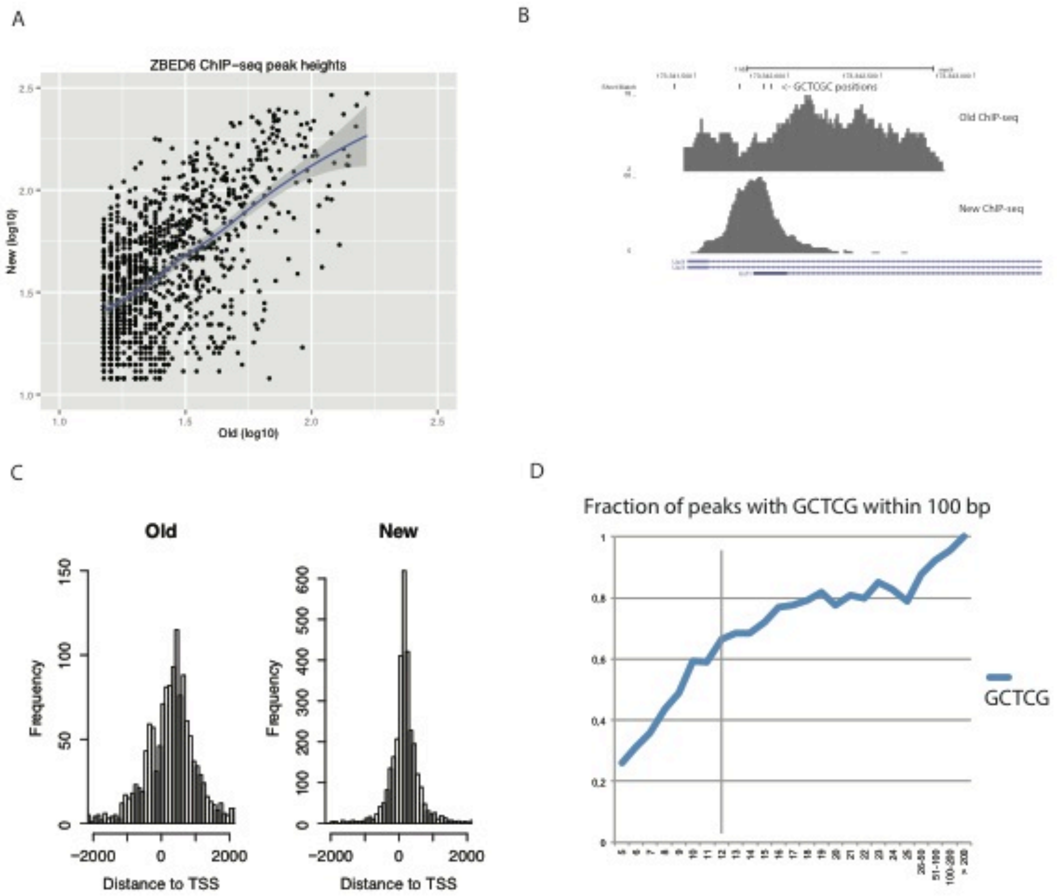

Figure S3. Results of ZBED6 ChIP-seq analysis in C2C12 cells. A) Comparison between peak heights for overlapping peaks from the old (x-axis) and new (y-axis) datasets. Despite lower sequencing depth the peaks were generally higher in the new replicate. B) Example of a gene (Usf1) which was called as a ZBED6 targets only in the new replicate. C) Peaks from both replicates are enriched downstream of TSS but the new data show a more focused distribution. D) Most of the peaks in the new dataset have a match to the consensus GCTCG sequence close to the peak summit. Vertical line at peak height 12 indicates the significance level used for comparison to DE genes.
