# Supplementary material for: ZBED6 Modulates the Transcription of Myogenic Genes in Mouse Myoblast Cells
Source: PLoS One. 2014 Apr 8;9(4):e94187. doi: 10.1371/journal.pone.0094187 (PMC3979763; doi:10.1371/journal.pone.0094187)
Supplement: Figure S4 — Validation of ZBED6 binding to the conserved element upstream of Twist2 . (PDF) [file pone.0094187.s004.pdf]

A

B

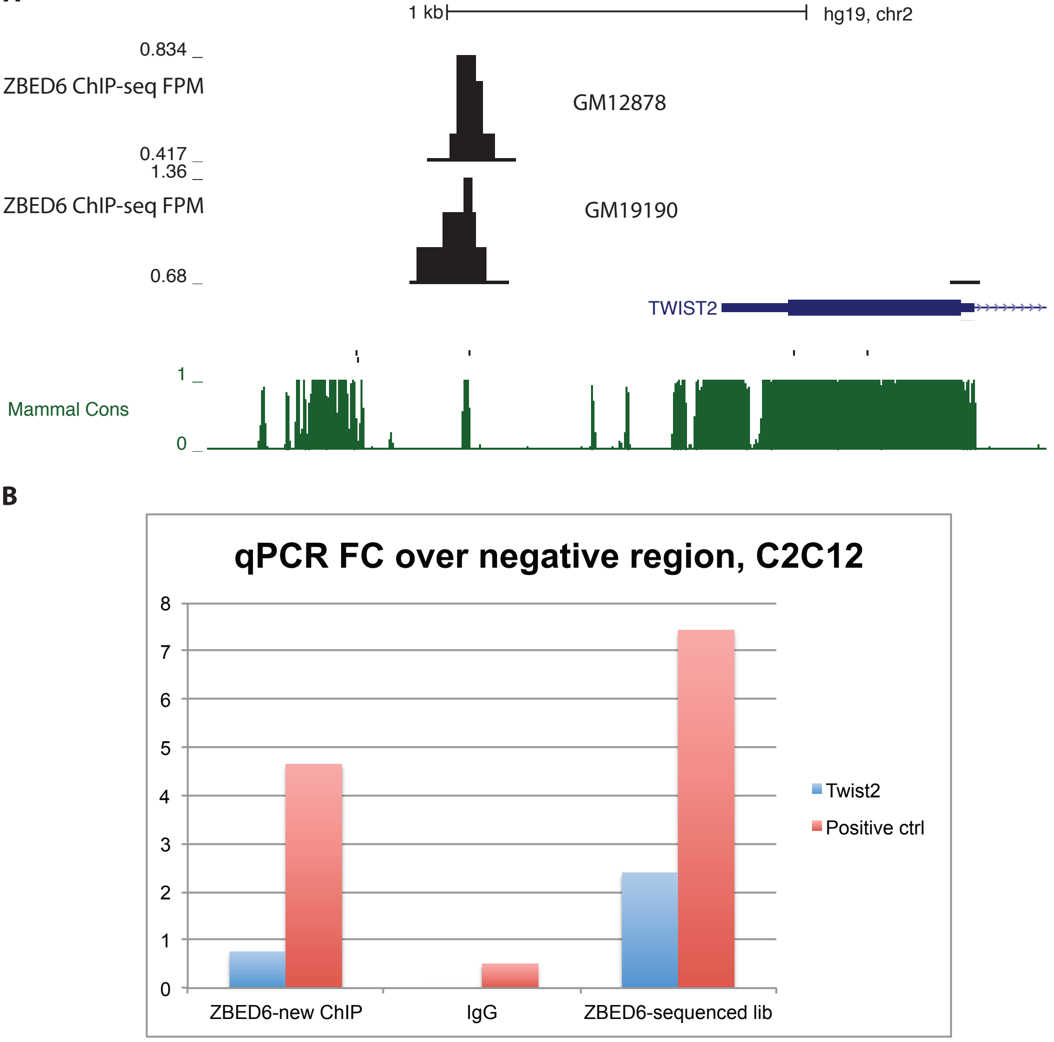

Figure S4. Validation of ZBED6 binding to the conserved element upstream of *Twist2.* A) Enrichment of ZBED6 ChIP-seq reads over the conserved element in two human cell lines (unpublished data). B) Quantitative PCR using an independent C2C12 ChIP sample and using the latest sequenced C2C12 ChIP-seq library show both enrichment for the *Twist2* site (blue). Fold change values (log2 scale) were computed against a negative control region, and the enrichment for one of the most highly enriched sites (red) was used as a positive control for the ChIP.
