# Supplementary material for: ZBED6 Modulates the Transcription of Myogenic Genes in Mouse Myoblast Cells
Source: PLoS One. 2014 Apr 8;9(4):e94187. doi: 10.1371/journal.pone.0094187 (PMC3979763; doi:10.1371/journal.pone.0094187)
Supplement: Figure S5 — Enrichment analysis of six histone modifications across ZBED6 target sites. ZBED6 target genes were divided into up-regulated and down-regulated genes after Zbed6 silencing. (PDF) [file pone.0094187.s005.pdf]

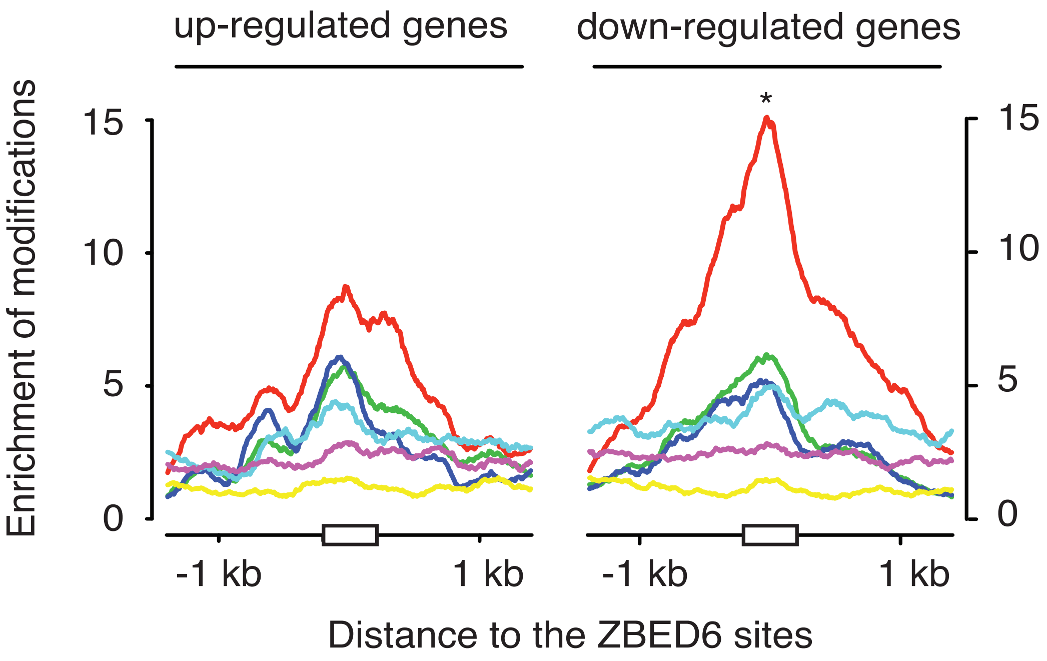

Figure S5. Enrichment analysis of six histone modifications across ZBED6 target sites. ZBED6 target genes were divided into up-regulated and down-regulated genes after Zbed6 silencing. The asterisk indicates a significant difference ($P<0.05$). H3K4me2 (dark red), H3K4me3 (green), H3K27ac (purple), H3K4me1 (blue), H3K27me3 (pink), and H3K36me3 (yellow).
