# Supplementary material for: ZBED6 Modulates the Transcription of Myogenic Genes in Mouse Myoblast Cells
Source: PLoS One. 2014 Apr 8;9(4):e94187. doi: 10.1371/journal.pone.0094187 (PMC3979763; doi:10.1371/journal.pone.0094187)
Supplement: Table S1 — Summary of RNA sequencing and reads mapping. (PDF) [file pone.0094187.s006.pdf]

Table S1. Summary of RNA sequencing and reads mapping.

Pooled samples:	Day 2 Control	Day 2 *Zbed6*-silenced	Day 4 Control	Day 4 *Zbed6*-silenced
Total reads (million)	49	51	53	42
Aligned reads (%)	51.7%	55.9%	57.8%	63.5%
Uniquely aligned (%)	43.2%	47.8%	48.1%	53.5%
