# Supplementary material for: ZBED6 Modulates the Transcription of Myogenic Genes in Mouse Myoblast Cells
Source: PLoS One. 2014 Apr 8;9(4):e94187. doi: 10.1371/journal.pone.0094187 (PMC3979763; doi:10.1371/journal.pone.0094187)
Supplement: Table S2 — Differentially expressed genes identified by RNA-seq. (PDF) [file pone.0094187.s007.pdf]

Table S2. Differentially expressed genes identified by RNA-seq. M-value is equal to log2 fold changes. N.A stands for not available.

Official gene symbol	Expression level (RPKM)	RNAseq Day2 M-value	RNAseq Day2 FDR	RNAseq Day4 M-value	RNAseq Day4 FDR	Array Day2 M-value	Array Day2 FDR
Ttn	7	1.0	0.0E+00	0.6	2.5E-141	0.0	0.94
Tpcn1	60	-1.3	0.0E+00	-0.7	7.7E-85	-0.6	0.0000
Tmbim6	180	-0.6	0.0E+00	-1.0	0.0E+00	-0.7	0.0000
Thbs1	169	0.9	0.0E+00	0.2	2.4E-129	-0.4	0.0000
Sh3glb1	191	0.9	0.0E+00	0.2	3.5E-114	0.4	0.0004
Sdpr	84	1.5	0.0E+00	1.3	0.0E+00	0.6	0.0000
Scd1	596	1.0	0.0E+00	0.2	0.0E+00	-0.2	0.22
Npnt	97	1.7	0.0E+00	1.0	0.0E+00	0.1	0.08
Mylpf	440	1.0	0.0E+00	0.9	1.9E-247	-0.2	0.40
Myh3	96	1.0	0.0E+00	0.8	3.5E-258	N.A.	N.A.
Ly6a	664	1.5	0.0E+00	0.5	3.9E-207	0.0	0.95
Lpp	33	1.1	0.0E+00	0.2	1.6E-80	N.A.	N.A.
Itgb1	659	0.8	0.0E+00	1.3	0.0E+00	0.0	0.70
H19	1349	0.8	0.0E+00	1.0	0.0E+00	0.3	0.07
Gsta4	448	-0.8	0.0E+00	-0.5	2.0E-45	-0.2	0.05
Gm11410	1678	0.7	0.0E+00	0.6	0.0E+00	N.A.	N.A.
Col18a1	66	-0.6	0.0E+00	-0.5	2.8E-25	-0.9	0.0000
Cfl1	629	-0.8	0.0E+00	-0.6	1.2E-115	N.A.	N.A.
AY036118	407	-1.4	0.0E+00	-1.6	5.0E-184	N.A.	N.A.
Actc1	201	1.4	0.0E+00	0.6	2.4E-55	0.3	0.019
Acta2	623	0.8	0.0E+00	0.9	0.0E+00	-0.1	0.62
Krt80	63	1.4	4.0E-304	0.5	9.1E-119	0.0	0.99
Basp1	168	-0.9	2.5E-285	-0.3	1.2E-05	0.0	0.65
Gm10222	2085	0.9	3.1E-267	1.7	0.0E+00	N.A.	N.A.
Vcl	245	0.6	4.6E-258	0.1	4.9E-69	-0.2	0.13
Gm13339	1315	0.9	2.7E-254	1.2	0.0E+00	N.A.	N.A.
Hk1	39	-0.7	1.9E-243	-1.0	1.1E-174	N.A.	N.A.
Mfap3l	115	0.9	1.0E-227	1.2	0.0E+00	0.0	0.96
Fndc3a	30	1.1	2.4E-216	0.0	8.3E-14	N.A.	N.A.
Pkia	51	1.4	2.2E-213	0.7	1.2E-118	0.7	0.0000
Sfrp2	40	-1.1	1.0E-208	-1.3	1.8E-15	-0.6	0.0000
Ppic	120	-1.1	3.9E-206	-1.9	0.0E+00	-0.8	0.0000
Sema3d	32	1.1	3.1E-199	0.6	8.0E-76	N.A.	N.A.
Igf2	28	1.7	6.5E-198	1.9	0.0E+00	0.3	0.04
Qk	93	0.7	5.4E-194	0.5	0.0E+00	N.A.	N.A.
Mir5115	1017	-1.6	8.0E-193	-3.6	0.0E+00	N.A.	N.A.
Cald1	158	0.7	1.3E-185	0.1	4.6E-81	0.0	0.58
Rod1	44	1.2	6.5E-180	0.3	3.6E-95	N.A.	N.A.
Ap3m1	96	0.7	2.7E-179	0.3	3.3E-151	0.0	1.00
Ccdc141	21	1.0	4.1E-176	0.4	1.7E-58	N.A.	N.A.
Samd8	30	1.2	8.4E-172	0.6	9.6E-146	0.1	0.26
Mir5105	798	-1.4	1.3E-171	-1.6	9.5E-62	N.A.	N.A.
Atp13a3	87	0.8	7.1E-162	0.5	0.0E+00	N.A.	N.A.
Csnk1g2	77	-0.7	3.3E-150	-1.1	3.0E-135	-0.3	0.06

There are more than 700 genes that did not show due to the limited space and the complete list is in the excel file.
