# Supplementary material for: ZBED6 Modulates the Transcription of Myogenic Genes in Mouse Myoblast Cells
Source: PLoS One. 2014 Apr 8;9(4):e94187. doi: 10.1371/journal.pone.0094187 (PMC3979763; doi:10.1371/journal.pone.0094187)
Supplement: Table S3 — qPCR validation of 12 differentially expressed genes that were identified by RNA-seq. (PDF) [file pone.0094187.s008.pdf]

Table S3. qPCR validation of 12 differentially expressed genes that were identified by RNA-seq.

Gene	Expression level (RPKM)	Expression categories	RNAseq Day2 M-value	RNAseq Day2 FDR	qPCR Day2 M-value	qPCR Day2 FDR
Fgf11	5	low	0.94	0.00	1.01	0.04
Nfkbil1	7	low	-0.75	0.00	-1.73	0.01
Myo5a	15	low	0.82	0.00	1.18	0.02
Nr4a1	31	medium	-0.76	0.00	-1.02	0.05
Sfrp2	40	medium	-1.20	0.00	-1.24	0.01
Ddit4	52	medium	-0.86	0.00	-0.61	0.32
Snord95	189	high	0.67	0.00	-0.28	0.56
Snord57	282	high	1.11	0.00	0.64	0.03
Snord12	371	high	1.00	0.00	-0.1	0.74
H19	1349	high	0.81	0.000	0.51	0.020
Snord82	1545	high	0.85	0.00	0.59	0.01
Snord47	4251	high	1.01	0.00	0.14	0.27
