# Supplementary material for: ZBED6 Modulates the Transcription of Myogenic Genes in Mouse Myoblast Cells
Source: PLoS One. 2014 Apr 8;9(4):e94187. doi: 10.1371/journal.pone.0094187 (PMC3979763; doi:10.1371/journal.pone.0094187)
Supplement: Table S4 — Gene Ontology analysis of genes with differential expressions identified by RNA-seq. (PDF) [file pone.0094187.s009.pdf]

Table S4. Gene Ontology analysis of genes with differential expressions identified by RNA-seq.

GO term	Count	PValue	Fold Enrichment	FDR
Muscle protein	13	5.0E-08	8.0	6.9E-05
Contractile fiber	16	2.0E-07	5.4	3.2E-04
Myofibril	15	8.0E-07	5.2	0.0011
Contractile fiber part	14	3.0E-06	5.2	0.0034
Cytoskeletal protein binding	33	5.0E-06	2.4	0.0080
Heart development	22	1.0E-05	3.0	0.0250
Muscle contraction	12	2.0E-05	5.6	0.0391
Actin cytoskeleton	20	2.0E-05	3.1	0.0326
Actin binding	25	2.0E-05	2.7	0.0364
Sarcomere	12	4.0E-05	4.8	0.0508
