# Supplementary material for: ZBED6 Modulates the Transcription of Myogenic Genes in Mouse Myoblast Cells
Source: PLoS One. 2014 Apr 8;9(4):e94187. doi: 10.1371/journal.pone.0094187 (PMC3979763; doi:10.1371/journal.pone.0094187)
Supplement: Table S5 — Twenty-six differentially expressed genes with evolutionary conserved ZBED6 binding sites. (PDF) [file pone.0094187.s010.pdf]

Table S5. Twenty-six differentially expressed genes with evolutionary conserved ZBED6 binding sites.

Gene symbol	RNAseq Day2 M-value	RNAseq Day2 FDR	Palindr-ome	Position	Palindrome and motif
Igf2	1.66	6.5E-198	yes	chr7:149,850,145-149,850,170	GCCTAGGCTCGC
Ppm1e	1.33	7.7E-20	yes	chr11:87,172,533-87,172,613	CCGAGCTCGG
Igsf11	1.11	2.5E-12	yes	chr16:38,902,446-38,902,529	TCCCGGGCTCGC
Epm2a	1.05	7.3E-05	yes	chr10:11,063,199-11,063,281	GGAGCTCGC
Fgf11	0.94	8.5E-14	yes	chr11:69,614,477-69,614,557	TCGAGCTCGC
Acvr2a	0.93	1.2E-30	yes	chr2:48,669,216-48,669,293	CGAGCTCGCTCG
Ankib1	0.90	1.1E-46	yes	chr5:3,802,755-3,802,836	TGAGCTCGC
Ppm1l	0.88	3.8E-22	yes	chr3:69,121,310-69,121,394	CGGCCGCTCG
Kbtbd8	0.84	8.6E-04	yes	chr6:95,068,245-95,068,327	GCCGGCTCG
Ablim3	0.83	4.6E-04	yes	chr18:62,071,395-62,071,471	AGAGCTCGC
Enho	0.83	5.4E-20	yes	chr4:41,587,326-41,587,406	GAGCTCG
Larp4	0.71	5.5E-41	yes	chr15:99,803,803-99,803,883	CACATGTTGCTCGC
Tmeff1	0.63	9.6E-08	No	chr4:48,598,746-48,598,833	GGGTTTGCTCG
G3bp2	0.62	7.0E-27	yes	chr5:92,512,633-92,512,716	CGAGCTCG
Homer1	0.58	1.1E-08	yes	chr13:94,073,557-94,073,589	AGAGCTCG
Arc	-0.60	7.9E-06	no	chr15:74,503,007-74,503,093	GGCAGCTCG
Socs3	-0.62	5.1E-21	no	chr11:117,829,280-117,829,370	GCGGGCTCG
Dlx3	-0.64	2.0E-16	no	chr11:94,981,609-94,981,698	TCCAGCTCGC
Slc9a3r1	-0.65	9.9E-18	no	chr11:115,025,314-115,025,393	CGCTGCTCG
Nfkbil1	-0.65	3.2E-09	no	chr17:35,372,053-35,372,138	CTGGGCTCGC
Pim3	-0.74	4.9E-46	no	chr15:88,693,848-88,693,934	CTGCGCTCG
Ier2	-0.80	3.1E-75	no	chr8:87,189,322-87,189,408	AGGGGCTCGC
Twist2	-0.80	3.8E-09	no	chr1:93,697,317-93,697,400	AGAGGCTCGC
Ddit4	-0.86	4.0E-146	no	chr10:59,414,122-59,414,207	CCAGGCTCGC
Stc2	-1.00	9.8E-92	no	chr11:31,269,956-31,270,039	CGCGGCTCG
Sfrp2	-1.12	1.0E-208	no	chr3:83,568,327-83,568,420	TCTCGCTCGC
