# Supplementary material for: ZBED6 Modulates the Transcription of Myogenic Genes in Mouse Myoblast Cells
Source: PLoS One. 2014 Apr 8;9(4):e94187. doi: 10.1371/journal.pone.0094187 (PMC3979763; doi:10.1371/journal.pone.0094187)
Supplement: Table S6 — Significant GO categories for the top 300 H3K9ac genes. (PDF) [file pone.0094187.s011.pdf]

Table S6. Significant GO categories for the top 300 H3K9ac genes.

Category	Term	Count	%	P-Value	Benjamini
**GOTERM_CC_FAT**	contractile fiber	17	5.7	2.4E-11	2.7E-09
**GOTERM_CC_FAT**	myofibril	17	5.7	1.5E-11	3.4E-09
**SP_PIR_KEYWORDS**	muscle protein	13	4.3	3.6E-11	9.4E-09
**GOTERM_CC_FAT**	sarcomere	15	5.0	5.7E-10	4.3E-08
**GOTERM_CC_FAT**	contractile fiber part	15	5.0	1.3E-09	7.5E-08
**GOTERM_MF_FAT**	calcium ion binding	31	10.3	5.9E-08	2.0E-05
**GOTERM_CC_FAT**	I band	10	3.0	1.2E-06	5.5E-05
**SP_PIR_KEYWORDS**	calcium	25	8.3	1.3E-06	1.8E-04
**GOTERM_CC_FAT**	actin cytoskeleton	14	4.7	4.9E-05	1.8E-03
**GOTERM_CC_FAT**	myosin complex	7	2.3	2.6E-04	8.4E-03
**GOTERM_CC_FAT**	Z disc	7	2.3	3.6E-04	1.0E-02
**KEGG_PATHWAY**	Cardiac muscle contraction	8	2.7	1.3E-04	1.3E-02
**KEGG_PATHWAY**	Hypertrophic cardiomyopathy (HCM)	8	2.7	2.9E-04	1.4E-02
**GOTERM_CC_FAT**	myosin filament	4	1.3	1.2E-03	3.0E-02
**GOTERM_BP_FAT**	muscle organ development	13	4.3	3.3E-05	3.7E-02
**INTERPRO**	Immunoglobulin subtype 2	9	3.0	5.6E-04	4.5E-02
**GOTERM_CC_FAT**	integral to plasma membrane	12	4.0	2.3E-03	5.0E-02
**INTERPRO**	Immunoglobulin I-set	8	2.7	5.2E-04	5.1E-02
**SP_PIR_KEYWORDS**	glycoprotein	54	18.0	6.2E-04	5.3E-02
**SMART**	IGc2	9	3.0	9.0E-04	5.5E-02
**INTERPRO**	Calcium-binding EF-hand	9	3.0	4.6E-04	5.6E-02
**INTERPRO**	EF-HAND 2	11	3.7	2.5E-04	6.0E-02
**KEGG_PATHWAY**	Dilated cardiomyopathy	7	2.3	1.8E-03	6.0E-02
**INTERPRO**	EF-HAND 1	11	3.7	4.0E-04	6.4E-02
**GOTERM_CC_FAT**	intrinsic to plasma membrane	12	4.0	3.5E-03	6.9E-02
**INTERPRO**	EF-Hand type	12	4.0	1.5E-04	7.3E-02
**SP_PIR_KEYWORDS**	skeletal muscle	4	1.3	1.2E-03	7.5E-02
**GOTERM_BP_FAT**	muscle contraction	7	2.3	3.0E-04	8.2E-02
**INTERPRO**	EGF-like calcium-binding. conserved site	7	2.3	1.6E-03	8.7E-02
**SMART**	EGF_CA	7	2.3	2.2E-03	8.7E-02
**INTERPRO**	EGF-like calcium-binding	7	2.3	1.5E-03	8.8E-02

**SMART**	EFh	9	3.0	7.5E-04	8.9E-02
**INTERPRO**	EGF-type aspartate/asparagine hydroxylation conserved site	7	2.3	1.3E-03	9.0E-02
**UP_SEQ_FEATURE**	domain:EF-hand 1	10	3.3	2.3E-04	9.3E-02
