# Supplementary material for: ZBED6 Modulates the Transcription of Myogenic Genes in Mouse Myoblast Cells
Source: PLoS One. 2014 Apr 8;9(4):e94187. doi: 10.1371/journal.pone.0094187 (PMC3979763; doi:10.1371/journal.pone.0094187)
Supplement: Table S7 — Primer and probe sets for qPCR validation. (PDF) [file pone.0094187.s012.pdf]

Table S7. Primer and probe sets for qPCR validation.

Gene	Primer/probe	Primer sequences (5'->3')
Zbed6	forward	CAAGACATCTGCAGTTTGGAATTT
	reverse	TGTCGTTGAAGTGTTGAAGTTCCTA
	probe	ACATCTCAAGAGCTGTGTGT
Igf2	forward	CGTGGCATCGTGGAAGAGT
	reverse	ACACGTCCCTCTCGGACTTG
	probe	CTGGCCCTCCTGGAG
18s	forward	AGTCCCTGCCCTTTGTACACA
	reverse	GATCCGAGGGCCTCACTAAAC
	probe	CGCCCGTCGCTACTACCGATTGG
Myog	forward	GGCTGCCTAAAGTGGAGATCCT
	reverse	AGGCCTGTAGG CGCTCAAT
	probe	CAGCGCCATCCAGT
Actb	forward	ATATCGCTGGGCTGGTCGTC
	reverse	AGGATGGCGTGAGGGAGAGC
H19	forward	AAGCTATCTCCGGGACTCCAA
	reverse	TTACCCCCTTTTGAATTTGCA
Sfrp2	forward	ACGACAACGACATCATGGAA
	reverse	ACGCCGTTCAGCTTGTAAAT
Ddit4	forward	GGTCTGCAGCCAGAGAAGAG
	reverse	GACACCCCATCCAGGTATGA
Nfkbil1	forward	ATGCCACCGGACCTGTTG
	reverse	GCCCCCCTCTGGAGGAA
Snord47	forward	CCAGTGATGTGATGATTCTGC
	reverse	AAATGGAACGGTTTAAAGGTG
Snord57	forward	GGAAAGGATGAACGAACTTGG
	reverse	CAGTTGTATTGCCTCCATTTCTG
Snord82	forward	ATTTCAACCCCATGGTTCAG
	reverse	CACAAGTGATGAGTGACAAAGG
Snord95	forward	GATGACCACAACATGCCATC
	reverse	AACAGCCTCTGGATTTCAGC
Snord12	forward	TTCTTTCCCCGTCAGATCG
	reverse	CATCAGACAAAACTGGCAATTAG
Nr4a1	forward	AGCACTGCCAAATTGGACTA
	reverse	TCTGCCCACTTTCGGATAAC
Myo5a	forward	ATGCCTACAGTGGCCAGAAC
	reverse	GCGCCTGACTCTCCACTTAC
Fgf11	forward	CAGGAGCTTGGGCACAAAG
	reverse	CATGAAGGGAAACCGAGTCAA
Prrx2	forward	AACCGCCGTGCCAAGTT
	reverse	CAGAGCGGGTAGCCAGCAT
Mef2a	forward	ACATGAACCAGGAAGCCTTAGG
	reverse	GCCCTTCTGTAAAACGCATGA
Elk4	forward	TCCGTCATCAAATTTGTGACAAC
	reverse	AGCAGCGACAGGCTCGAT
Sp1	forward	GGCTACCCCTACCTCAAAGGA
	reverse	TTGGAAGACTCGCTGCCATT
Nfkb1	forward	AGCAGGATGCTGAGGATTCTG

	reverse	GGCAACTCTGTCCTGCACCTA
